# Modeling the Role of Baseline Risk and Additional Study‐Level Covariates in Meta‐Analysis of Treatment Effects

**DOI:** 10.1002/sim.70278

**Published:** 2025-10-23

**Authors:** Phuc T. Tran, Annamaria Guolo

**Affiliations:** ^1^ Department of Statistical Sciences University of Padova Padova Italy

**Keywords:** baseline risk, heterogeneity, likelihood, measurement error, meta‐analysis

## Abstract

The relationship between the treatment effect and the baseline risk is a recognized tool to investigate the heterogeneity of treatment effects in meta‐analyses of clinical trials. Since the baseline risk is difficult to measure, a proxy is adopted, which is based on the rate of events for the subject under the control condition. The use of the proxy in terms of aggregated information at the study level implies that the data are affected by measurement errors, a problem that the literature has explored and addressed in recent years. This paper proposes an extension of the classical meta‐analysis with baseline risk information, which includes additional study‐specific covariates other than the rate of events to explain heterogeneity. Likelihood‐based inference is carried out by including measurement error correction techniques necessary to prevent unreliable inference due to the measurement errors affecting the covariates summarized at the study level. Within‐study covariances between risk measures and the covariate components are computed using Taylor expansions based on study‐level covariate subgroup summary information. When such information is not available and, more generally, in order to reduce computational difficulties, a pseudo‐likelihood solution is developed under a working independence assumption between the observed error‐prone measures. The performance of the methods is investigated in a series of simulation studies under different specifications for the sample size, the between‐study heterogeneity, and the underlying risk distribution. They are applied to a meta‐analysis about the association between COVID‐19 and schizophrenia.

## 1 Introduction

One common application of meta‐analysis is the evaluation of the effectiveness of a treatment, exploiting information from different studies comparing a treatment group and a control group [1, 2, 3]. A proper analysis needs to detect and account for between‐study heterogeneity, which occurs as a consequence of different sources, including differences in studies' protocols and design, execution, and experience of the staff, as well as differences in patients' characteristics and general health, such as age, gender, and severity of illness. If individual patient data are available from each study included in the meta‐analysis, then an accurate investigation of the relationship between treatment risk and features of patients and/or studies is possible. Unfortunately, information is usually available in terms of aggregated data at the study level, that is, in terms of summary results from published studies [4, 5]. Pioneering studies in the 1990s suggest that a relevant source of heterogeneity is represented by the underlying risk or the baseline risk of disease of study participants; see, for example, Sharp [2] and Schmid et al. [3]. This kind of information is not available at the patient level, and it is also difficult to measure, since it can be seen as an aggregation of relevant attributes of the patient and the severity of illness. Accordingly, the baseline risk is approximated by using a surrogate measure on patients under the control condition, that is, a measure of the rate at which the outcome of interest occurs. The inclusion of a study‐specific measure of the baseline risk for the controls in the meta‐analysis model in order to explain between‐study heterogeneity gives rise to a meta‐regression model [6, 7, 8]. Since the risk measured on controls is aggregated study‐level information used as a proxy for the true unknown baseline risk, it is affected by error [1, 7, 9, 10]. The presence of measurement error can affect inference and give rise to inconsistent results, as the first studies in McIntosh [1] and Sharp and Thompson [11] show. See also the discussion in Arends et al. [6], van Houwelingen et al. [7] and Guolo et al. [9]. More generally, outside the meta‐analysis framework, a substantial and long‐established literature has shown that the consequences of measurement errors in linear and nonlinear regression models can be substantial if errors are not corrected for. See the book‐length discussion in Carroll et al. [12], Yi [13], and Yi et al. [14]. The best known effect is attenuation bias, that is, a biased toward zero estimate of the coefficient associated with the risk measure in the control group, which occurs under an additive and homoscedastic between‐study error on the underlying risk measure; see, for example, van Houwelingen et al. [7] and Guolo [10]. Investigations in meta‐analysis, including a measure of the baseline risk, have provided several solutions to correct for the presence of measurement errors. Methods include structural and functional approaches, according to the choice between accepting assumptions on the underlying unobserved control measure (structural approaches) or not (functional approaches). See Ghidey et al. [15] and Guolo [10] for a comparison of some of the techniques, including likelihood‐based solutions under different assumptions of the underlying risk, corrected and conditional scores, and a simulation‐based approach [16].

Traditionally, control risk as a measure of the baseline risk is the only covariate considered in the meta‐regression model of treatment effectiveness to describe between‐study heterogeneity. This paper proposes an extension that includes additional study‐specific covariates other than the control risk, which might help to improve the explanation of heterogeneity in the treatment risk. Such additional covariates are summary quantities at the study level, for example, the mean age of patients or the percentage of females/males. As it happens, for the measure of the baseline risk, the available summarized covariates are a surrogate for the actual true measurement that would occur at the individual subject level in the study, and thus, they are likely affected by measurement errors. Appropriate inference should take this additional source of error, as well as the one associated with the baseline risk measure, into account. The paper focuses on a structural approach using likelihood‐based correction techniques applied to a hierarchical model relating the unobserved error‐prone quantities to their observed counterparts under an approximate or an exact measurement error specification. When the approximate normal error structure is chosen, mainly for computational convenience, within‐study covariances between risk measures and the covariate components are computed by using Taylor expansions based on study‐level covariate subgroup summary information. When such information is not available and, more generally, in order to reduce computational difficulties, a pseudo‐likelihood solution is developed under a working independence assumption between the observed error‐prone measures. Under the exact binomial or multinomial measurement error structure, the likelihood function has no closed form, and it can be computationally intensive to maximize. The pseudo‐likelihood version is considered as a viable option.

The performance of the proposed solutions is investigated in a series of simulation studies, including different types of additional covariates, either a mean response or a binary indicator of a feature from each study included in the meta‐analysis. Scenarios cover different specifications for sample size, between‐study heterogeneity, and underlying risk distribution. The applicability of the methods is also evaluated on a meta‐analysis of the association between mortality from COVID‐19 and schizophrenia.

## 2 Classical Meta‐Analysis Model With Baseline Risk Information

Consider a meta‐analysis of n independent studies comparing a treatment group and a control group in order to investigate the effectiveness of a treatment. Let $\eta_{i}$ and $\xi_{i}$ denote the measure of risk in the treatment group and in the control group for study i, respectively, as, for example, log‐odds or log‐event rate. The common meta‐analysis model, including baseline risk information, assumes that the quantities are related according to a linear model [6, 9],

(1) $$\begin{array}{l} \eta_{i}=\beta_{0}+\beta_{1}\xi_{i}+\varepsilon_{i},\;\;\varepsilon_{i}∼N\left(0,\tau^{2}\right), \end{array}$$

where $\tau^{2}$ is the residual variance describing the variation among studies in the treatment risk $\eta_{i}$ unexplained by the underlying risk $\xi_{i}$, for example, heterogeneity due to patient characteristics or study design. The sampling errors $\varepsilon_{i}$ are independent of the underlying risk $\xi_{i}$. The inferential interest is usually in $\beta_{1}$, which represents the relationship between $\eta_{i}$ and $\xi_{i}$. The use of the control risk as a proxy for the unknown and unavailable baseline risk requires a model to be specified to relate the true/unobserved quantities and the mismeasured counterparts. Since the information from study i is an average value thought to be representative of the subjects included in that study, the measurement error model is specified through a classical structure, according to the measurement error terminology [12, 14]. This means that the error is additive and homoscedastic, with mismeasured values thought to vary randomly around the unobserved true value. The specification of the exact measurement error structure is strictly related to the available information from each study included in the meta‐analysis. To fix the ideas, and without loss of generality, let $Y_{i}$ and $X_{i}$ denote the number of events in the treatment group and in the control group from study i, respectively, and let $n_{iT}$ and $n_{iC}$ denote the number of treated and the number of controls, respectively. Using the log‐odds as a risk measure, the observed error‐prone version of ${\left(\eta_{i},\xi_{i}\right)}^{T}$ is given by

(2) $${\hat{\eta }}_{i}=\log\left(\frac{Y_{i}}{n_{iT}-Y_{i}}\right),\;\;{\hat{\xi }}_{i}=\log\left(\frac{X_{i}}{n_{iC}-X_{i}}\right)$$

with associated standard error

(3) $$s_{\eta_{i}}^{2}=\frac{1}{Y_{i}}+\frac{1}{n_{iT}-Y_{i}},\;\;s_{\xi_{i}}^{2}=\frac{1}{X_{i}}+\frac{1}{n_{iC}-X_{i}}$$

According to an approximate specification, the relationship between ${\left({\hat{\eta }}_{i},{\hat{\xi }}_{i}\right)}^{T}$ and ${\left(\eta_{i},\xi_{i}\right)}^{T}$ is directly modeled, usually through a normal specification for computational convenience [1, 6],

$$\left(\begin{array}{c} {\hat{\eta }}_{i}\\ {\hat{\xi }}_{i} \end{array}\right)|\left(\begin{array}{c} \eta_{i}\\ \xi_{i} \end{array}\right)∼N\left(\left(\begin{array}{c} \eta_{i}\\ \xi_{i} \end{array}\right),\left(\begin{array}{cc} s_{\eta_{i}}^{2} & 0\\ 0 & s_{\xi_{i}}^{2} \end{array}\right)\right).$$

The exact measurement error model relating ${\left(Y_{i},X_{i}\right)}^{T}$ to ${\left(\eta_{i},\xi_{i}\right)}^{T}$ can be specified by considering the binomial distribution for $Y_{i}$ and $X_{i}$ [4, 6], namely,

$$\begin{array}{lll} Y_{i}|\eta_{i}∼\text{Binomial}\left\{n_{iT},\text{expit}(\eta_{i})\right\}, & \; & \\ X_{i}|\xi_{i}∼\text{Binomial}\left\{n_{iC},\text{expit}(\xi_{i})\right\}. & & \end{array}$$

See, for example, Guolo [9] for details and different specifications, such as, for example, the Poisson case.

Following the terminology of the measurement error literature, in this paper, we will consider a structural approach to deal with error‐prone measures of ${\left(\eta_{i},\xi_{i}\right)}^{T}$. Accordingly, the unobserved mismeasured quantities are interpreted as random variables, and a distribution function for them needs to be specified. Assume that the underlying risk $\xi_{i}$ follows a Normal distribution, $\xi_{i}∼N\left(\mu_{\xi },\sigma_{\xi }^{2}\right)$, mainly for computational convenience. Accordingly, under the approximate measurement error model, the marginal distribution for ${\left({\hat{\eta }}_{i},{\hat{\xi }}_{i}\right)}^{T}$ is

$$\left(\begin{array}{c} {\hat{\eta }}_{i}\\ {\hat{\xi }}_{i} \end{array}\right)∼N_{2}\left\{\left(\begin{array}{c} \beta_{0}+\beta_{1}\mu_{\xi }\\ \mu_{\xi } \end{array}\right),\left(\begin{array}{cc} s_{\eta_{i}}^{2}+\beta_{1}^{2}\sigma_{\xi }^{2}+\tau^{2} & \beta_{1}\sigma_{\xi }^{2}\\ \beta_{1}\sigma_{\xi }^{2} & s_{\xi_{i}}^{2}+\sigma_{\xi }^{2} \end{array}\right)\right\},$$

and the likelihood function for the whole parameter vector $\theta ={\left(\beta_{0},\beta_{1},\mu_{\xi },\tau^{2},\sigma_{\xi }^{2}\right)}^{⊤}$ has a closed‐form expression,

L(θ)∝∏i=1ndetsηi2+β12σξ2+τ2β1σξ2β1σξ2sξi2+σξ2−12 ϕ2sηi2+β12σξ2+τ2β1σξ2β1σξ2sξi2+σξ2−12η^iξ^i−β0+β1μξμξ,

where $\phi_{2}$ denotes the density function of the bivariate standard normal distribution.

In case the exact measurement error model is specified, the likelihood function associated with the number of events ${\left(Y_{i},X_{i}\right)}^{T}$ is not in closed‐form,

(4) L(θ)∝∏i=1n∫−∞+∞∫−∞+∞expitηiYi1−expitηiniT−Yi expitξiXi1−expitξiniC−Xi ×1τ2ϕηi−β0−β1ξiτ×1σξ2ϕξi−μξσξdηidξi,

where ϕ denotes the density function of the standard normal distribution. The computation of the likelihood function can be done by using numerical methods, for example, Gauss–Hermite quadrature. Inference based on the exact measurement error model can be prone to some issues. Several authors warn against the risk of convergence problems, given by a nonpositive definite covariance matrix and values of the variance components on the boundary of the parameter space, especially in the case of small sample size [17, 18, 19].

## 3 Including Additional Covariates

This paper investigates the extension of the classical meta‐analysis model with baseline risk information to include study‐specific covariates as a way to explain potential residual heterogeneity. The focus will be on a study‐specific covariate $\zeta_{i}$, which may or may not be affected by measurement error. This will give rise to different modifications of the meta‐regression model and its measurement error component and, as a consequence, to modifications of the likelihood expression.

### 3.1 Error‐Free Covariates

Suppose that each study included in the meta‐analysis provides information about features of the study or patients characteristics that are not affected by errors. Typically, such information is not a summarized quantity over the subjects included in the study; instead, it is a common quantity, for example, the country where the study has been conducted. In this case, $\zeta_{i}$ only enters the model with baseline risk information (1),

(5) $$\begin{array}{l} \eta_{i}=\beta_{0}+\beta_{1}\xi_{i}+\beta_{2}\zeta_{i}+\varepsilon_{i},\;\;\xi_{i}∼N\left(0,\sigma_{\xi }^{2}\right),\;\;\varepsilon_{i}∼N\left(0,\tau^{2}\right), \end{array}$$

while the measurement error structure is not affected. Correspondingly, there is no additional computational effort in the likelihood‐based analysis.

### 3.2 Error‐Affected Covariates

More often, the additional covariate represents aggregated information over the subjects included in each study, for example, the mean age or the percentage of females/males. The study‐specific information ${\hat{\zeta }}_{i}$ is a summary of information of the true unknown covariate $\zeta_{i}$, and thus it is affected by errors. The measurement error model relating ${\left({\hat{\eta }}_{i},{\hat{\xi }}_{i}\right)}^{T}$ to ${\left(\eta_{i},\xi_{i}\right)}^{T}$ must be extended to include a distribution for ${\hat{\zeta }}_{i}$ given the true unknown quantity. Following the structural approach for measurement error correction, a marginal distribution for $\zeta_{i}$ must be specified. Choosing a Normal distribution $\zeta_{i}∼N\left(\mu_{\zeta },\sigma_{\zeta }^{2}\right)$, for computational convenience, the model for risk measures and the error‐affected covariate can be expressed as

(6) $$\begin{array}{l} \eta_{i}=\beta_{0}+\beta_{1}\xi_{i}+\beta_{2}\zeta_{i}+\varepsilon_{i}, \end{array}$$

with

$$\begin{array}{l} \xi_{i}∼N\left(\mu_{\xi },\sigma_{\xi }^{2}\right),\;\;\zeta_{i}∼N\left(\mu_{\zeta },\sigma_{\zeta }^{2}\right),\;\;\varepsilon_{i}∼N\left(0,\tau^{2}\right). \end{array}$$

For the moment, suppose we work with the approximate error model (2). Inserting the information from ${\hat{\zeta }}_{i}$ in the approximate error model leads to

(7) $$\left.\left(\begin{array}{c} {\hat{\eta }}_{i}\\ {\hat{\xi }}_{i}\\ {\hat{\zeta }}_{i} \end{array}\right)\;\right|\;\left(\begin{array}{c} \eta_{i}\\ \xi_{i}\\ \zeta_{i} \end{array}\right)∼N_{3}\left(\left(\begin{array}{c} \eta_{i}\\ \xi_{i}\\ \zeta_{i} \end{array}\right),\Gamma_{i}=\left(\begin{array}{ccc} s_{\eta_{i}}^{2} & 0 & s_{\eta_{i},\zeta_{i}}\\ 0 & s_{\xi_{i}}^{2} & s_{\xi_{i},\zeta_{i}}\\ s_{\eta_{i},\zeta_{i}} & s_{\xi_{i},\zeta_{i}} & s_{\zeta_{i}}^{2} \end{array}\right)\right),$$

where $s_{\zeta_{i}}^{2}$ denotes the within‐study variance of ${\hat{\zeta }}_{i}$, $s_{\eta_{i},\zeta_{i}}$ denotes the within‐study covariance between ${\hat{\eta }}_{i}$ and ${\hat{\zeta }}_{i}$, and $s_{\xi_{i},\zeta_{i}}$ denotes the within‐study covariance between ${\hat{\xi }}_{i}$ and ${\hat{\zeta }}_{i}$. The marginal distribution for the observed measures of risk and covariate is

(8) $$\left(\begin{array}{c} {\hat{\eta }}_{i}\\ {\hat{\xi }}_{i}\\ {\hat{\zeta }}_{i} \end{array}\right)∼N_{3}(\mu ,\sum +\Gamma_{i}),$$

where

$$\begin{array}{ll} \mu & =\left(\begin{array}{c} \beta_{0}+\beta_{1}\mu_{\xi }+\beta_{2}\mu_{\zeta }\\ \mu_{\xi }\\ \mu_{\zeta } \end{array}\right),\sum =\left(\begin{array}{ccc} \beta_{1}^{2}\sigma_{\xi }^{2}+\beta_{2}^{2}\sigma_{\zeta }^{2}+\tau^{2} & \beta_{1}\sigma_{\xi }^{2} & \beta_{2}\sigma_{\zeta }^{2}\\ \beta_{1}\sigma_{\xi }^{2} & \sigma_{\xi }^{2} & \sigma_{\xi \zeta }\\ \beta_{2}\sigma_{\zeta }^{2} & \sigma_{\xi \zeta } & \sigma_{\zeta }^{2} \end{array}\right). \end{array}$$

Let $\theta ={\left(\beta_{0},\beta_{1},\beta_{2},\mu_{\xi },\mu_{\zeta },\tau^{2},\sigma_{\xi }^{2},\sigma_{\zeta }^{2},\sigma_{\xi \zeta }\right)}^{⊤}$ be the whole parameter vector from model (8). Given the normality assumptions, the likelihood function for θ has a closed‐form expression,

(9) $$\begin{array}{ll} L\left(\theta \right) & \propto \prod_{i=1}^{n}{\left\{\det\left(\sum +\Gamma_{i}\right)\right\}}^{-\frac{1}{2}}\phi_{3}\left[{\left(\sum +\Gamma_{i}\right)}^{-\frac{1}{2}}\left\{\left(\begin{array}{c} {\hat{\eta }}_{i}\\ {\hat{\xi }}_{i}\\ {\hat{\zeta }}_{i} \end{array}\right)-\mu \right\}\right], \end{array}$$

where $\phi_{3}$ denotes the density function of a three‐dimensional standard normal variable. It can be assumed that $s_{\zeta_{i}}^{2}$ is known and equal to its estimator $\hat{var}\left({\hat{\zeta }}_{i}|\zeta_{i}\right)$ provided that the sample size is large enough. However, the within‐study variance might be unknown if the information about the additional covariate is given only in terms of the mean values. In this case, it can be replaced by a value that is based on previous studies or expert opinion. As an alternative, consider that subgroup summary information is likely to be available from studies included in the meta‐analysis. It can be exploited to derive an approximation of the within‐study covariances. The following subsections discuss how to obtain the approximation of the within‐study covariances in two cases, namely, when the covariate $\zeta_{i}$ is expressed in terms of log odds and when the covariate $\zeta_{i}$ is the mean value of a characteristic over the studies.

#### 3.2.1 Approximation of Within‐Study Covariances When the Covariate is a Log Odds

Suppose that each study includes information about the number of subjects within each subgroup of events/no events identified by an additional discrete covariate. To fix the ideas, consider a binary indicator $Z_{i}$ about the number of males/females within the event/no event cases for the treatment group and the control group, as illustrated in Table 1.

**TABLE 1:** Subgroup summary information for a binary indicator $Z_{i}$.

Treatment group				Control group			
Event		Non‐event		Event		Non‐event	
Male	Female	Male	Female	Male	Female	Male	Female
$Z_{iT1M}$	$Z_{iT1F}$	$Z_{iT0M}$	$Z_{iT0F}$	$Z_{iC1M}$	$Z_{iC1F}$	$Z_{iC0M}$	$Z_{iC0F}$

The number of events in the treatment group and the control group can be computed as

(10) $$\begin{array}{l} Y_{i}=Z_{iT1M}+Z_{iT1F},\;\;X_{i}=Z_{iC1M}+Z_{iC1F}, \end{array}$$

respectively. Let $Z_{i}$ denote the number of male patients, and let subgroups be identified by subscripts, so that subscripts M and F indicate female/male, subscripts T and C indicate the treatment group/control group, and subscripts 1 and 0 indicate event/no‐event, respectively. For example, $Z_{iT1M}$ denotes the number of treated male patients with the event. Accordingly,

$$Z_{i}=Z_{iT1M}+Z_{iT0M}+Z_{iC1M}+Z_{iC0M}.$$

Assume that the male indicator at the individual level is distributed as a Bernoulli variable with success probability $\text{expit}\left(\zeta_{i}\right)$,

(11) $$Z_{i}|\zeta_{i}∼\text{Binomial}\left\{n_{i},\text{expit}\left(\zeta_{i}\right)\right\}$$

where $n_{i}$ denotes the sample size in study i, $n_{i}=n_{iT}+n_{iC}$. Accordingly, $\zeta_{i}$ is the unobserved log odds of male, whose observed error‐prone version ${\hat{\zeta }}_{i}$ and its associated variance $s_{\zeta_{i}}^{2}$ are

(12) $$\begin{array}{l} {\hat{\zeta }}_{i}=\log\frac{Z_{i}}{n_{i}-Z_{i}},\;\;s_{\zeta_{i}}^{2}=\frac{1}{Z_{i}}+\frac{1}{n_{i}-Z_{i}}, \end{array}$$

respectively. In this case, it is possible to compute the within‐study covariances between the observed risk measures and $Z_{i}$. Using the first‐order Taylor expansion [20], it can be shown that

(13) $$\begin{array}{lll} s_{\eta_{i},\zeta_{i}} & \approx \; & \frac{1}{Z_{i}}\left(\frac{Z_{iT1M}}{Y_{i}}-\frac{Z_{iT0M}}{n_{iT}-Y_{i}}\right)-\frac{1}{n_{i}-Z_{i}}\left(\frac{Z_{iT1F}}{Y_{i}}-\frac{Z_{iT0F}}{n_{iT}-Y_{i}}\right), \end{array}$$

and

(14) $$\begin{array}{lll} s_{\xi_{i},\zeta_{i}} & \approx \; & \frac{1}{Z_{i}}\left(\frac{Z_{iC1M}}{X_{i}}-\frac{Z_{iC0M}}{n_{iC}-X_{i}}\right)-\frac{1}{n_{i}-Z_{i}}\left(\frac{Z_{iC1F}}{X_{i}}-\frac{Z_{iC0F}}{n_{iC}-X_{i}}\right). \end{array}$$

See the Appendix for details.

#### 3.2.2 An Approximation to Within‐Study Covariances When the Covariate is a Mean Value

Suppose that each study includes information about the mean of a covariate $Z_{i}$ measured on the subjects, subdivided by group (treatment/control) and event (event/no‐event). For example, $Z_{i}$ may represent the mean age of patients in the treatment/control group, subdivided by event/no‐event cases. The subgroup summary information can be shown in Table 2, where subscripts T, C, 1, and 0 have the same interpretation as before.

**TABLE 2:** Subgroup summary information when $Z_{i}$ is the mean value per study.

Treatment group		Control group	
Event	Non‐event	Event	Non‐event
$Z_{iT1}$	$Z_{iT0}$	$Z_{iC1}$	$Z_{iC0}$

Accordingly,

$$Z_{i}=\frac{Y_{i}Z_{iT1}+\left(n_{iT}-Y_{i}\right)Z_{iT0}+X_{i}Z_{iC1}+\left(n_{iC}-X_{i}\right)Z_{iC0}}{n_{i}}.$$

Assuming that the unobserved covariate at the individual level follows a Normal distribution with mean $\zeta_{i}$ and variance $SD_{i}^{2}$, $Z_{i}$ has a conditional Normal distribution

(15) $$\begin{array}{l} Z_{i}|\zeta_{i}∼N\left(\zeta_{i},\frac{SD_{i}^{2}}{n_{i}}\right), \end{array}$$

so that

(16) $$\begin{array}{l} {\hat{\zeta }}_{i}=Z_{i},\;\;s_{\zeta_{i}}^{2}=\frac{SD_{i}^{2}}{n_{i}}. \end{array}$$

The covariances between the observed measures of risk and the observed covariate $Z_{i}$ can be approximated via the first‐order Taylor expansion [20], leading to

(17) $$\begin{array}{lll} s_{\eta_{i},\zeta_{i}} & \approx \; & \frac{1}{n_{i}}(Z_{iT1}-Z_{iT0}),\;\;s_{\xi_{i},\zeta_{i}}\approx \frac{1}{n_{i}}(Z_{iC1}-Z_{iC0}). \end{array}$$

See the Appendix for details.

#### 3.2.3 Pseudo‐Likelihood Approach Under Approximate Error Model

The log‐likelihood function associated with the model, including the additional covariate information $Z_{i}$, whichever the expression of $Z_{i}$, can give rise to issues in evaluation and maximization when study‐specific information is not available. We mainly refer to the covariance components of the within‐study covariance matrix. When available study‐specific information is not among those in Tables 1, 2, a different approach is proposed which relies on the concept of pseudo‐likelihood [21]. According to this view, we suggest replacing the likelihood function by a pseudo version, developed under the working conditional independence assumption of ${\hat{\eta }}_{i},{\hat{\xi }}_{i}$ and ${\hat{\zeta }}_{i}$, which sets to zero the unknown covariances $s_{\eta_{i}\zeta_{i}},s_{\xi_{i}\zeta_{i}}$. Starting from the approximate measurement error model (7), the pseudo‐likelihood function for θ is

$$\begin{array}{l} pL\left(\theta \right)\propto \prod_{i=1}^{n}{\left\{\det\left(\sum +{\tilde{\Gamma }}_{i}\right)\right\}}^{-\frac{1}{2}}\phi_{3}\left[{\left(\sum +{\tilde{\Gamma }}_{i}\right)}^{-\frac{1}{2}}\left\{\left(\begin{array}{c} {\hat{\eta }}_{i}\\ {\hat{\xi }}_{i}\\ {\hat{\zeta }}_{i} \end{array}\right)-\mu \right\}\right], \end{array}$$

where

$$\begin{array}{l} {\tilde{\Gamma }}_{i}=\left(\begin{array}{ccc} s_{\eta_{i}}^{2} & 0 & 0\\ 0 & s_{\xi_{i}}^{2} & 0\\ 0 & 0 & s_{\zeta_{i}}^{2} \end{array}\right). \end{array}$$

In order to account for the risk of misspecification of the likelihood components, the covariance matrix of the estimators can be conveniently computed by using the sandwich formula [22], which is available in the Appendix.

#### 3.2.4 Pseudo‐Likelihood Approach Under Exact Measurement Error Models

As for the classical meta‐analysis model with baseline risk information, in the case of study‐specific covariates, the exact measurement error model can be specified in place of the approximate error model. A likelihood function can be derived by marginalizing the joint density function of the outcomes, the true measures of risk, and the additional covariate. For example, when the additional covariate is a log odds and subgroup summary information is given in terms of Table 1, the subgroup outcomes can be modeled using multinomial distributions

(18) $$\begin{array}{ll} & {\left(Z_{iT1M},Z_{iT0M},Z_{iT1F},Z_{iT0F}\right)}^{⊤}|{\left(\eta_{i},\zeta_{i}\right)}^{⊤}\\ & \;∼{\text{Multinomial}}_{4}\left(p_{iT1M},p_{iT0M},p_{iT1F},p_{iT0F}\right),\\ & {\left(Z_{iC1M},Z_{iC0M},Z_{iC1F},Z_{iC0F}\right)}^{⊤}|{\left(\xi_{i},\zeta_{i}\right)}^{⊤}\\ & \;∼{\text{Multinomial}}_{4}\left(p_{iC1M},p_{iC0M},p_{iC1F},p_{iC0F}\right), \end{array}$$

where vectors of probabilities ${\left(p_{iT1M},p_{iT0M},p_{iT1F},p_{iT0F}\right)}^{⊤}$ and ${\left(p_{iC1M},p_{iC0M},p_{iC1F},p_{iC0F}\right)}^{⊤}$ are functions of ${\left(\eta_{i},\zeta_{i}\right)}^{⊤}$ and ${\left(\xi_{i},\zeta_{i}\right)}^{⊤}$, respectively,

$$\begin{array}{ll} p_{iT1M}+p_{iT1F} & =\text{expit}(\eta_{i})\\ p_{iT1M}+p_{iT0M} & =\text{expit}(\zeta_{i})\\ p_{iT1M}+p_{iT0M}+p_{iT1F}+p_{iT0F} & =1\\ p_{iC1M}+p_{iC1F} & =\text{expit}(\xi_{i})\\ p_{iC1M}+p_{iC0M} & =\text{expit}(\zeta_{i})\\ p_{iC1M}+p_{iC0M}+p_{iC1F}+p_{iC0F} & =1. \end{array}$$

In this case, the likelihood function for θ has a more complex expression if compared to the case of the approximate measurement error model, namely,

$$\begin{array}{ll} L(\theta ) & \propto \overset{n}{\underset{i=1}{\prod }}\int_{-\infty }^{\infty }\int_{-\infty }^{\infty }\int_{-\infty }^{\infty }p_{iT1M}^{Z_{iT1M}}\times p_{iT0M}^{Z_{iT0M}}\times p_{iT1F}^{Z_{iT1F}}\times p_{iT0F}^{Z_{iT0F}}\\ & \times p_{iC1M}^{Z_{iC1M}}\times p_{iC0M}^{Z_{iC0M}}\times p_{iC1F}^{Z_{iC1F}}\times p_{iC0F}^{Z_{iC0F}}\times \frac{1}{\sqrt{\tau^{2}}}\times \frac{1}{\sqrt{\sigma_{\xi }^{2}}}\times \frac{1}{\sqrt{\sigma_{\zeta }^{2}}}\\ & \times \phi \left(\frac{\eta_{i}-\beta_{0}-\beta_{1}\xi_{i}-\beta_{2}\zeta_{i}}{\tau }\right)\phi \left(\frac{\xi_{i}-\mu_{\xi }}{\sigma_{\xi }}\right)\phi \left(\frac{\zeta_{i}-\mu_{\zeta }}{\sigma_{\zeta }}\right)d\eta_{i}d\xi_{i}d\zeta_{i}. \end{array}$$

Such a likelihood function does not have a closed‐form expression and is very expensive to maximize, since every integrand is a product of many functions involving ${\left(\eta_{i},\xi_{i},\zeta_{i}\right)}^{⊤}$.

When the additional covariate is the mean response of a characteristic and subgroup summary information is given in terms of Table 2, the subgroup outcomes given $X_{i}$ and $Y_{i}$ can be modeled using bivariate Normal distributions, that is,

(19) $$\begin{array}{ll} \left(\begin{array}{c} Z_{iT1}\\ Z_{iT0} \end{array}\right)|\left(\begin{array}{c} Y_{i}\\ \zeta_{i} \end{array}\right) & ∼N_{2}\left(\left(\begin{array}{c} \zeta_{i}\\ \zeta_{i} \end{array}\right),\left\{\begin{array}{cc} \frac{SD_{i}^{2}}{Y_{i}} & \frac{\rho_{iT}SD_{i}^{2}}{\sqrt{Y_{i}\left(n_{iT}-Y_{i}\right)}}\\ \frac{\rho_{iT}SD_{i}^{2}}{\sqrt{Y_{i}\left(n_{iT}-Y_{i}\right)}} & \frac{SD_{i}^{2}}{n_{iT}-Y_{i}} \end{array}\right\}\right),\\ \left(\begin{array}{c} Z_{iC1}\\ Z_{iC0} \end{array}\right)|\left(\begin{array}{c} X_{i}\\ \zeta_{i} \end{array}\right) & ∼N_{2}\left(\left(\begin{array}{c} \zeta_{i}\\ \zeta_{i} \end{array}\right),\left\{\begin{array}{cc} \frac{SD_{i}^{2}}{X_{i}} & \frac{\rho_{iC}SD_{i}^{2}}{\sqrt{X_{i}\left(n_{iC}-X_{i}\right)}}\\ \frac{\rho_{iC}SD_{i}^{2}}{\sqrt{X_{i}\left(n_{iC}-X_{i}\right)}} & \frac{SD_{i}^{2}}{n_{iC}-X_{i}} \end{array}\right\}\right) \end{array}$$

where $\rho_{iT}$ and $\rho_{iC}$ denote within‐study correlations which are functions of $Y_{i}$ and $X_{i}$, respectively. Again, the associated likelihood function has a complex form,

$$\begin{array}{ll} L(\theta ) & \propto \overset{n}{\underset{i=1}{\prod }}\int_{-\infty }^{+\infty }\int_{-\infty }^{+\infty }\int_{-\infty }^{+\infty }\phi_{2}\\ & \;\left[{\left\{\begin{array}{cc} \frac{SD_{i}^{2}}{Y_{i}} & \frac{\rho_{iT}SD_{i}^{2}}{\sqrt{Y_{i}\left(n_{iT}-Y_{i}\right)}}\\ \frac{\rho_{iT}SD_{i}^{2}}{\sqrt{Y_{i}\left(n_{iT}-Y_{i}\right)}} & \frac{SD_{i}^{2}}{n_{iT}-Y_{i}} \end{array}\right\}}^{-\frac{1}{2}}\left\{\left(\begin{array}{c} Z_{iT1}\\ Z_{iT0} \end{array}\right)-\left(\begin{array}{c} \zeta_{i}\\ \zeta_{i} \end{array}\right)\right\}\right]\\ & \times \phi_{2}\left[{\left\{\begin{array}{cc} \frac{SD_{i}^{2}}{X_{i}} & \frac{\rho_{iC}SD_{i}^{2}}{\sqrt{X_{i}\left(n_{iC}-X_{i}\right)}}\\ \frac{\rho_{iC}SD_{i}^{2}}{\sqrt{X_{i}\left(n_{iC}-X_{i}\right)}} & \frac{SD_{i}^{2}}{n_{iC}-X_{i}} \end{array}\right\}}^{-\frac{1}{2}}\left\{\left(\begin{array}{c} Z_{iC1}\\ Z_{iC0} \end{array}\right)-\left(\begin{array}{c} \zeta_{i}\\ \zeta_{i} \end{array}\right)\right\}\right]\\ & \times {\left\{\text{expit}\left(\eta_{i}\right)\right\}}^{Y_{i}}{\left\{1-\text{expit}\left(\eta_{i}\right)\right\}}^{n_{iT}-Y_{i}}{\left\{\text{expit}\left(\xi_{i}\right)\right\}}^{X_{i}}\\ & \;{\left\{1-\text{expit}\left(\xi_{i}\right)\right\}}^{n_{iC}-X_{i}}\\ & \times \frac{1}{\sqrt{\tau^{2}}}\phi \left(\frac{\eta_{i}-\beta_{0}-\beta_{1}\xi_{i}-\beta_{2}\zeta_{i}}{\tau }\right)\frac{1}{\sqrt{\sigma_{\xi }^{2}}}\phi \left(\frac{\xi_{i}-\mu_{\xi }}{\sigma_{\xi }}\right)\\ & \;\frac{1}{\sqrt{\sigma_{\zeta }^{2}}}\phi \left(\frac{\zeta_{i}-\mu_{\zeta }}{\sigma_{\zeta }}\right)d\eta_{i}d\xi_{i}d\zeta_{i}. \end{array}$$

When subgroup summary information is unavailable, the construction of the likelihood function can be more cumbersome. While the number of events $Y_{i}$ and $X_{i}$ are independent as measured on different subjects, this is not true when $Z_{i}$ is taken into account, as it typically refers to characteristics of the subjects ($Y_{i}$ and $X_{i}$) included in each study. In addition, a distribution for $Z_{i}$ given the unobserved $\zeta_{i}$ must be specified as well as a marginal distribution for $\zeta_{i}$, according to the structural approach for error correction. Let $f_{Y_{i},X_{i},Z_{i}|\eta_{i},\xi_{i},\zeta_{i}}$ denote the density function associated with the distribution of $Y_{i},X_{i},Z_{i}|\eta_{i},\xi_{i},\zeta_{i}$. Let $f_{\eta_{i}|\xi_{i}\zeta_{i}}$ denote the density function from model (6), and let $f_{\xi_{i}|\zeta_{i}}$ and $f_{\zeta_{i}}$ denote the density functions associated with $\xi_{i}|\zeta_{i}$ and $\zeta_{i}$, respectively. An extension of likelihood function (4) to include the uncertainty associated with the additional study‐specific covariate $Z_{i}$ makes the integral three‐dimensional, with increasing computational issues,

$$L(\theta )=\overset{n}{\underset{i=1}{\prod }}\int_{-\infty }^{+\infty }\int_{-\infty }^{+\infty }\int_{-\infty }^{+\infty }f_{Y_{i},X_{i},Z_{i}|\eta_{i},\xi_{i},\zeta_{i}}f_{\eta_{i}|\xi_{i}\zeta_{i}}f_{\xi_{i}|\zeta_{i}}f_{\zeta_{i}}d\eta_{i}d\xi_{i}d\zeta_{i},$$

where the dependence of the functions in the integrand on the parameters of interest is suppressed for convenience of notation.

Deriving the expression of the density functions for $Y_{i},X_{i}$ and $Z_{i}$ conditional of ${\left(\zeta_{i},\xi_{i},\zeta_{i}\right)}^{⊤}$ can be difficult. More generally, the evaluation of L(θ) can be cumbersome given the amount of conditional densities, also in terms of the accuracy measures with the additional covariate component $\zeta_{i}$. In order to reduce these obstacles, a pseudo‐likelihood solution can be adopted, which assumes conditional independence between the observed $Y_{i}$ and $X_{i}$ and the covariate $Z_{i}$ within each study as well as independence between the underlying risk $\xi_{i}$ and $\zeta_{i}$. See the Appendix for pseudo‐likelihood functions when the covariate is a log odds and when the covariate is a mean value. The evaluation of the integrals requires numerical integration, for example, using Gauss–Hermite quadrature. As for the previous case in Section 3.2.3, the sandwich formula is suggested to compute the covariance matrix of the maximum pseudo‐likelihood estimators.

## 4 Simulation Study

The likelihood and the pseudo‐likelihood approaches have been evaluated in a series of simulation studies covering different scenarios, including additional study‐specific covariates. The approaches are compared with the naive analysis, which ignores the presence of measurement errors. Data are generated under the following scenarios:

Scenario 1
Model (5) with an error‐free covariate $\zeta_{i}∼N\left(0,1\right)$;
Scenario 2
Model (6) with an error‐affected covariate, which is a binary indicator of a characteristic as in (18);
Scenario 3
Model (6) with an error‐affected covariate, which is the mean response of a characteristic from each study as in (19).

The code for simulation is written in the R programming language [23] and it is available as https://github.com/davidtran2908/control_risk_regression2.

Data are simulated according to the following procedure. First, for a given number n of studies included in the meta‐analysis, the number of treated $n_{iT}$ and the number of controls $n_{iC}$ in each study are generated from a uniform distribution $U\left(15,200\right)$. The true risk measures ${\left(\eta_{i},\xi_{i}\right)}^{T}$ and the study‐level information $\zeta_{i}$ are generated from model (5) for scenario 1 and model (6) for scenarios 2 and 3. Values of ${\left(Y_{i},X_{i}\right)}^{T}$ are then obtained. For scenario 1, these measures are from a binomial distribution (2). For scenario 2, ${\left(Y_{i},X_{i}\right)}^{T}$ are computed based on formula (10) after the generation of subgroup summary information ${\left(Z_{iT1M},Z_{iT1F},Z_{iT0M},Z_{iT0F},Z_{iC1M},Z_{iC1F},Z_{iC0M},Z_{iC0F}\right)}^{⊤}$, i=1,…,n, from model (18), with probabilities $p_{iT1M}$ and $p_{iC1M}$ simulated from uniform distributions $U(0,\min\left\{\text{expit}\left(\eta_{i}\right),\text{expit}\left(\zeta_{i}\right)\right\})$ and $U\left(0,\min\left\{\text{expit}\left(\xi_{i}\right),\text{expit}\left(\zeta_{i}\right)\right\}\right)$, respectively. For scenario 3, ${\left(Y_{i},X_{i}\right)}^{T}$ are derived from a binomial distribution (2) and ${\left(Z_{iT1},Z_{iT0},Z_{iC1},Z_{iC0}\right)}^{⊤}$ are generated from model (19), with within‐study correlations $\rho_{iT}$ and $\rho_{iC}$ in model (19) generated from a uniform distribution U(−1,1). At the final step, the observed error‐prone quantities ${\left({\hat{\eta }}_{i},{\hat{\xi }}_{i}\right)}^{T}$ are obtained using (2) and the associated standard errors are derived using (3). The observed error‐prone ${\hat{\zeta }}_{i}$ and the associated standard error follow formulas (12) and (16) for scenarios 2 and 3, respectively. Within‐study covariances are given as in ((13), (14)) for scenario 2 and as in (17) for scenario 3.

Values for the underlying risk $\xi_{i}$ are generated from a standard normal distribution, $\xi_{i}∼N(0,1)$, and from a skew‐normal distribution [24], $\xi_{i}∼SN(0,1,-5)$, in order to investigate the performance of the approaches in case of departures from the normality assumptions. Regression parameters are set equal to ${\left(\beta_{0},\beta_{1},\beta_{2}\right)}^{⊤}={\left(0.0,1.0,0.8\right)}^{⊤}$. Increasing values of the variance component is considered, namely, $\tau^{2}\in \{0.1,0.5,1.0\}$, as well as increasing values of the number of studies included in the meta‐analysis, n∈{10,20}. One thousand datasets are generated for each scenario and each combination of sample size n, between‐study variance $\tau^{2}$, and control risk $\xi_{i}$ distribution. Likelihood maximization, based on the Nelder–Mead algorithm [25], employs the ordinary least squares estimate as a starting value. Numerical integration is performed through Gauss–Hermite quadrature with 10 nodes.

The competing methods are examined in terms of bias, standard error (se), and standard deviation (sd) of the estimator of ${\left(\beta_{0},\beta_{1},\beta_{2},\mu_{\xi },\mu_{\zeta },\tau^{2},\sigma_{\xi }^{2},\sigma_{\zeta }^{2}\right)}^{⊤}$, and number of convergent solutions (conv). The empirical coverage probability is computed for 95% confidence intervals of ${\left(\beta_{0},\beta_{1},\beta_{2}\right)}^{⊤}$ using the Hessian standard error and the sandwich standard error.

### 4.1 Simulation Results

**FIGURE 1:**
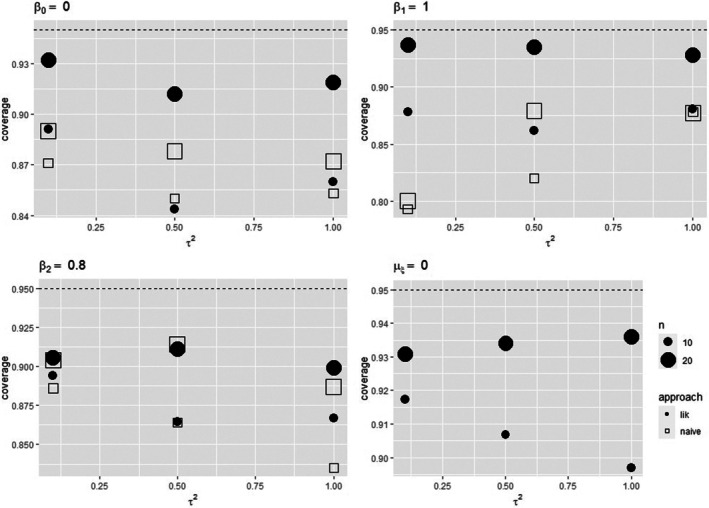
Empirical coverage probabilities of 95% Wald‐type confidence intervals for ${\left(\beta_{0},\beta_{1},\beta_{2},\mu_{\xi }\right)}^{⊤}$ for the uncorrected approach and the likelihood approach when data follow scenario 1. Underlying risk distributed as a standard Normal.

Table 3 reports the bias, the standard error and the standard deviation of the maximum likelihood estimator and the naive estimator of the parameters, when data are generated from model (5) with an additional error‐free covariate $\zeta_{i}∼N\left(0,1\right)$ and normally distributed control risk. Not surprisingly, results from the likelihood approach tend to be more satisfactory than the uncorrected solution, with a less biased estimator of the parameter of interest $\beta_{1}$. The behavior is more evident in the case of increasing values of the between‐study variance $\tau^{2}$. Figure 1 reports the empirical coverage probabilities of 95% Wald‐type confidence intervals for ${\left(\beta_{0},\beta_{1},\beta_{2}\right)}^{⊤}$, showing that results from the likelihood approach are closer to the target level than those from the naive analysis, with results improving as the number of studies grows. Similar results are obtained under an error‐free additional covariate when moving to a skewed control risk distribution or to model (5) with $\zeta_{i}∼\text{Bernoulli}\left(0.5\right)$. See Tables S1, S12, and S13 and Figures S1, S4, and S5 in the Supporting Information.

**TABLE 3:** Bias, standard error (se), standard deviation (sd) of the maximum likelihood estimators of ${\left(\beta_{0},\beta_{1},\beta_{2},\mu_{\xi },\tau^{2},\sigma_{\xi }^{2}\right)}^{⊤}$ and numbers of convergent solutions over 1000 replicates for the uncorrected approach and the likelihood approach when data follow scenario 1. Underlying risk distributed as a standard Normal.

			n=10				n=20			
$\tau^{2}$	Parameter	Approach	bias	se	sd	convergence	bias	se	sd	convergence
0.1	$\beta_{0}$	lik	0.007	0.134	0.160	991	−0.006	0.096	0.102	998
		naive	0.019	0.149	0.176	1000	−0.003	0.104	0.121	1000
	$\beta_{1}$	lik	0.011	0.172	0.214	991	0.012	0.121	0.136	998
		naive	−0.032	0.164	0.227	1000	−0.061	0.107	0.147	1000
	$\beta_{2}$	lik	−0.014	0.134	0.160	991	−0.010	0.119	0.128	998
		naive	0.014	0.152	0.180	1000	0.014	0.140	0.157	1000
	$\mu_{\xi }$	lik	−0.010	0.302	0.314	991	0.016	0.219	0.224	998
	$\tau^{2}$	lik	−0.056	0.048	0.062	982	−0.036	0.050	0.055	996
		naive	0.091	0.072	0.125	1000	0.120	0.064	0.090	1000
	$\sigma_{\xi }^{2}$	lik	−0.088	0.440	0.447	991	−0.072	0.320	0.322	998
0.5	$\beta_{0}$	lik	−0.000	0.232	0.295	998	0.001	0.165	0.182	1000
		naive	−0.004	0.340	0.405	1000	−0.008	0.179	0.214	1000
	$\beta_{1}$	lik	0.019	0.282	0.363	998	0.012	0.195	0.213	1000
		naive	−0.027	0.293	0.376	1000	−0.042	0.187	0.218	1000
	$\beta_{2}$	lik	−0.023	0.195	0.247	998	−0.017	0.186	0.204	1000
		naive	0.014	0.280	0.342	1000	0.023	0.222	0.248	1000
	$\mu_{\xi }$	lik	−0.002	0.299	0.319	998	−0.006	0.217	0.221	1000
	$\tau^{2}$	lik	−0.217	0.188	0.233	994	−0.128	0.161	0.171	1000
		naive	0.114	0.230	0.403	1000	0.094	0.173	0.217	1000
	$\sigma_{\xi }^{2}$	lik	−0.106	0.434	0.462	998	−0.093	0.315	0.307	1000
1	$\beta_{0}$	lik	0.009	0.296	0.374	999	−0.011	0.251	0.273	1000
		naive	0.009	0.346	0.432	1000	−0.006	0.258	0.315	1000
	$\beta_{1}$	lik	−0.003	0.381	0.472	999	−0.007	0.254	0.275	1000
		naive	−0.046	0.408	0.474	1000	−0.058	0.248	0.296	1000
	$\beta_{2}$	lik	−0.012	0.434	0.520	999	−0.009	0.285	0.327	1000
		naive	0.024	0.455	0.576	1000	0.016	0.311	0.380	1000
	$\mu_{\xi }$	lik	−0.005	0.293	0.328	999	−0.005	0.218	0.223	1000
	$\tau^{2}$	lik	−0.365	0.358	0.411	996	−0.251	0.290	0.314	1000
		naive	−0.035	0.361	0.539	1000	0.066	0.311	0.397	1000
	$\sigma_{\xi }^{2}$	lik	−0.143	0.417	0.441	999	−0.088	0.317	0.318	1000

Table 4 and Tables S2 and S3 in Supporting Information report the bias, the standard error, and the standard deviation of the likelihood estimators and the pseudo‐likelihood estimators of the parameters in model (6), when data include an error‐affected covariate, namely, a binary indicator of a characteristic of the patients, as in model (18). The pseudo‐likelihood approach and the likelihood approach based on subgroup summary have similar and satisfactory performance, with almost unbiased estimators. The improvement over the naive analysis in terms of estimate of $\beta_{1}$ becomes relevant when the number of studies increases.

**TABLE 4:** Bias, standard error (se), standard deviation (sd) of the maximum likelihood estimators of ${\left(\beta_{0},\beta_{1},\beta_{2},\mu_{\xi },\mu_{\zeta },\tau^{2},\sigma_{\xi }^{2},\sigma_{\zeta }^{2}\right)}^{⊤}$ and number of convergent solutions over 1000 replicates for the uncorrected approach, the likelihood approach and the pseudo‐likelihood approach when data follow scenario 2 and $\tau^{2}=0.1$. Underlying risk is distributed as a standard Normal.

			n=10				n=20			
$\tau^{2}$	Parameter	Approach	bias	se	sd	convergence	bias	se	sd	convergence
0.1	$\beta_{0}$	lik	0.000	0.150	0.166	992	0.007	0.102	0.109	997
		pseudo‐likelihood	−0.001	0.151	0.167	988	0.007	0.104	0.109	994
		naive	−0.002	0.161	0.184	1000	0.008	0.112	0.125	1000
	$\beta_{1}$	lik	0.004	0.185	0.203	992	0.009	0.126	0.131	997
		pseudo‐likelihood	0.006	0.186	0.206	988	0.008	0.126	0.130	994
		naive	−0.036	0.169	0.211	1000	−0.064	0.111	0.139	1000
	$\beta_{2}$	lik	−0.012	0.168	0.197	991	−0.003	0.113	0.124	997
		pseudo‐likelihood	−0.012	0.171	0.197	988	−0.001	0.115	0.125	994
		naive	−0.015	0.171	0.211	1000	−0.002	0.116	0.139	1000
	$\mu_{\xi }$	lik	0.008	0.302	0.309	993	−0.001	0.217	0.225	997
		pseudo‐likelihood	0.012	0.303	0.309	988	−0.003	0.219	0.229	994
	$\mu_{\zeta }$	lik	0.002	0.299	0.315	993	0.000	0.215	0.222	997
		pseudo‐likelihood	0.004	0.300	0.319	988	0.001	0.216	0.224	994
	$\tau^{2}$	lik	−0.046	0.060	0.066	979	−0.030	0.054	0.056	987
		pseudo‐likelihood	−0.047	0.059	0.066	978	−0.031	0.054	0.058	984
		naive	0.099	0.075	0.124	1000	0.147	0.072	0.107	1000
	$\sigma_{\xi }^{2}$	lik	−0.090	0.444	0.451	993	−0.089	0.316	0.318	997
		pseudo‐likelihood	−0.083	0.446	0.447	988	−0.079	0.320	0.320	994
	$\sigma_{\zeta }^{2}$	lik	−0.087	0.428	0.423	993	−0.079	0.305	0.306	997
		pseudo‐likelihood	−0.079	0.431	0.424	988	−0.069	0.308	0.308	994

**FIGURE 2:**
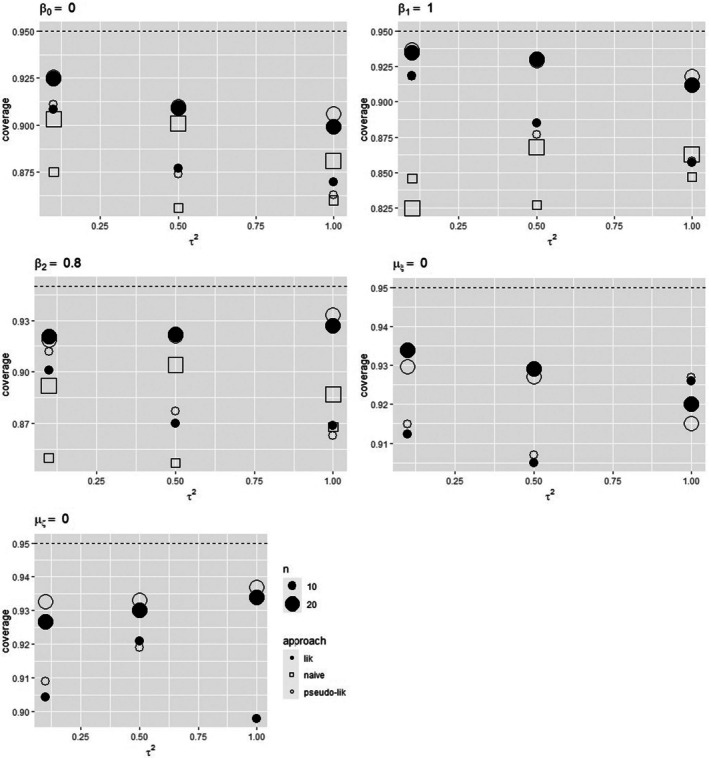
Empirical coverage probabilities of 95% Wald‐type confidence intervals for ${\left(\beta_{0},\beta_{1},\beta_{2},\mu_{\xi },\mu_{\zeta }\right)}^{⊤}$ for the uncorrected approach, the likelihood approach, and the pseudo‐likelihood approach when data follow scenario 2. Underlying risk distributed as a standard Normal.

Specifically, the estimators of the regression coefficients are nearly unbiased. Although the between‐study variance $\tau^{2}$ is underestimated, the relative bias of its estimator declines in magnitude with the number of studies and the residual variance. The standard errors of the estimators of ${\left(\beta_{0},\beta_{1},\beta_{2},\tau^{2}\right)}^{⊤}$ reduce when increasing n, as expected, as well as their differences with respect to the associated standard deviations. The empirical coverage probabilities of 95% Wald‐type confidence intervals for ${\left(\beta_{0},\beta_{1},\beta_{2}\right)}^{⊤}$ from the likelihood‐based solutions are comparable (see Figure 2) and notably outperform the ones from the uncorrected approach, whichever the values of the sample size and the between‐study variance. For both the likelihood and the pseudo‐likelihood approach, the empirical coverage probabilities tend to increase toward the nominal level as n increases and the between‐study heterogeneity decreases.

**FIGURE 3:**
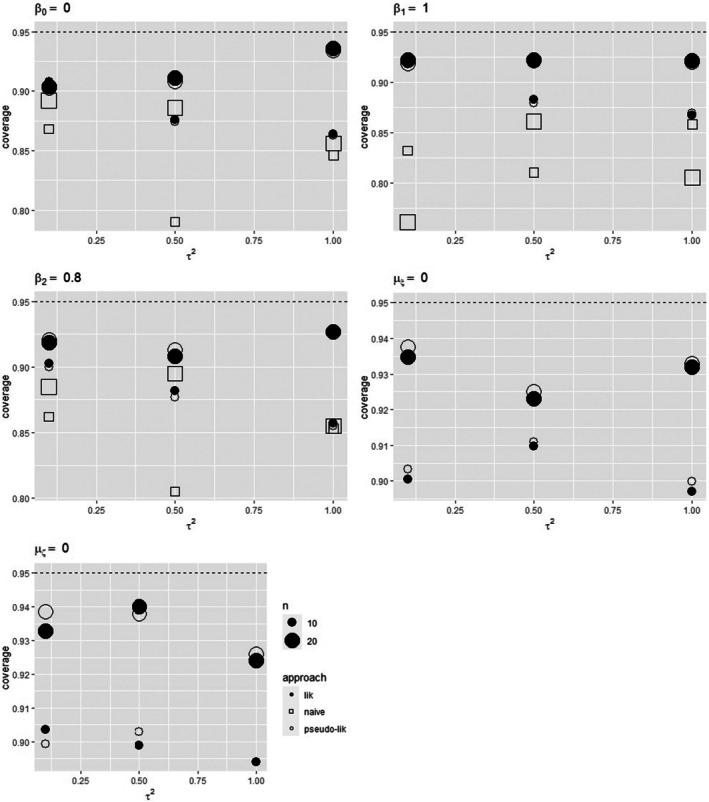
Empirical coverage probabilities of 95% Wald‐type confidence intervals for ${\left(\beta_{0},\beta_{1},\beta_{2},\mu_{\xi },\mu_{\zeta }\right)}^{⊤}$ for the uncorrected approach, the likelihood approach, and the pseudo‐likelihood approach when data follow scenario 3. Underlying risk distributed as a standard Normal.

When data include an error‐affected covariate defined as a mean value, following model (6) and model (19), the results from the competing approaches are similar to those experienced for data with $\zeta_{i}$ obtained as a binary indicator. See Table 5 for $\tau^{2}=0.1$, Tables S4, S5 in the Supporting Information for $\tau^{2}=0.5$ and $\tau^{2}=1.0$, respectively, and Figure 3.

**TABLE 5:** Bias, standard error (se), standard deviation (sd) of the maximum likelihood estimators of ${\left(\beta_{0},\beta_{1},\beta_{2},\mu_{\xi },\mu_{\zeta },\tau^{2},\sigma_{\xi }^{2},\sigma_{\zeta }^{2}\right)}^{⊤}$ and number of convergent solutions over 1000 replicates for the uncorrected approach, the likelihood approach and the pseudo‐likelihood approach when data follow scenario 3 and $\tau^{2}=0.1$. Underlying risk distributed as a standard Normal.

			n=10				n=20			
$\tau^{2}$	Parameter	Approach	bias	se	sd	convergence	bias	se	sd	convergence
0.1	$\beta_{0}$	lik	−0.004	0.148	0.170	995	0.001	0.104	0.118	996
		pseudo‐lik	−0.004	0.148	0.169	992	0.001	0.104	0.118	994
		naive	−0.005	0.160	0.185	1000	0.003	0.114	0.134	1000
	$\beta_{1}$	lik	0.008	0.185	0.216	995	0.006	0.129	0.143	996
		pseudo‐lik	0.009	0.186	0.216	992	0.007	0.129	0.144	994
		naive	−0.047	0.170	0.225	1000	−0.084	0.111	0.148	1000
	$\beta_{2}$	lik	−0.001	0.165	0.191	995	−0.012	0.111	0.124	996
		pseudo‐lik	−0.002	0.165	0.192	992	−0.013	0.111	0.123	994
		naive	0.017	0.176	0.225	1000	0.011	0.120	0.144	1000
	$\mu_{\xi }$	lik	0.005	0.299	0.326	995	−0.001	0.220	0.229	996
		pseudo‐lik	0.005	0.299	0.323	992	−0.001	0.220	0.228	994
	$\mu_{\zeta }$	lik	0.008	0.293	0.322	995	0.003	0.216	0.217	996
		pseudo‐lik	0.006	0.294	0.325	992	0.002	0.216	0.215	994
	$\tau^{2}$	lik	−0.045	0.059	0.069	985	−0.033	0.054	0.056	984
		pseudo‐lik	−0.046	0.059	0.069	987	−0.034	0.054	0.056	981
		naive	0.102	0.075	0.135	1000	0.147	0.072	0.100	1000
	$\sigma_{\xi }^{2}$	lik	−0.109	0.436	0.441	995	−0.080	0.325	0.311	996
		pseudo‐lik	−0.107	0.437	0.450	992	−0.080	0.325	0.316	994
	$\sigma_{\zeta }^{2}$	lik	−0.093	0.411	0.445	995	−0.049	0.304	0.308	996
		pseudo‐lik	−0.092	0.411	0.441	992	−0.051	0.303	0.306	994

When control risk follows an asymmetric distribution, $\xi_{i}∼SN(0,1,-5)$, results from the naive analysis are much worse than those under the Normal case, especially when data are generated from model (6) and model (19). See Tables S6–S8 in the Supporting Material. Improvements given by the likelihood‐based solutions are thus more evident, although they also suffer from an increased bias of the estimators of the regression parameters and of the variance components. Bias reduces as the sample size increases. A larger difference between the standard error and standard deviation of the estimators is an expected indication of model misspecification, with evidence for small sample size and large between‐study variance, as well as a slightly reduced coverage of confidence intervals; see Figure S2 in the Supporting Information.

The application of the likelihood approach and of the pseudo‐likelihood version does not pose computational problems. The failure rate is smaller than 3% in settings characterized by small sample size and small between‐study variance, especially when using the likelihood solution in place of the pseudo‐likelihood version.

### 4.2 A Simulation Study for the Exact Pseudo‐Likelihood Approach

The previous simulation study has focused on the approximate measurement error model, mainly for computational convenience. Under an exact measurement error model, in fact, the likelihood function is quite complex, as shown in Section 3.2.4. However, in order to investigate the behavior of the pseudo‐likelihood approach under an exact measurement error model, a small simulation study has been performed. The simulation study refers to data generated with an additional covariate represented by a binary indicator, as in model (18), with ${\left(n,\tau^{2}\right)}^{⊤}={\left(10,0.5\right)}^{⊤}$. When evaluating the pseudo‐likelihood function using Gaussian–Hermite quadrature, the number of nodes in each dimension is set equal to 10. The simulation is based on 100 replicates, for computational reasons. Results are reported in Table S14 in the Supporting Information.

These results confirm the previous findings under the approximate measurement error model. The pseudo‐likelihood approach provides estimators of the parameters with small bias, especially when focusing on regression coefficients. The slight differences between the standard errors and the associated standard deviations are expected since we are adopting the pseudo‐likelihood solution. The empirical coverage probabilities associated to the regression coefficients estimators are lower than the nominal level. This gap can be recovered by increasing the number of nodes in the numerical evaluation of integrals involved in the pseudo‐likelihood function.

## 5 Application: Schizophrenia and COVID‐19

Populations with older age, unhealthy lifestyle, physical comorbidities, and psychiatric diseases might be easily affected by COVID‐19 [26, 27, 28]. The literature indicates that there exist risk factors in schizophrenic patients associated with an increased risk of getting worse impacts of COVID‐19 [29], as for example, a dysregulated immune response [30, 31]. Pardamean and colleagues [28] conducted a meta‐analysis of ten studies to investigate the relationship between schizophrenia and mortality due to COVID‐19. The data, reported in Table 6, include the numbers of deaths (events) and the total number of observed subjects for the group with schizophrenia and the group without schizophrenia. Additional information from each study includes the mean age, the percentage of male patients, and the percentage of diabetic patients. Figure S6 in the Supporting Information reports a forest plot of the data, highlighting a substantial heterogeneity among the studies [34, 39]. Pardamean and colleagues [28] consider a meta‐regression model for the risk ratio including several risk factors. Results from the restricted maximum likelihood approach indicate that schizophrenic patients have a higher risk of mortality due to COVID‐19 compared to patients without schizophrenia (RR=2.22; 95% CI: (1.54,3.20)). Associations between mortality in schizophrenic patients and mean age $\left({\hat{\beta }}_{\text{age}}=-0.033\text{; 95\% CI:}\;\left(-0.052,-0.015\right)\right)$ and the percentage of smokers $\left({\hat{\beta }}_{\text{smoker}}=0.027\text{; 95\% CI:}\;\left(0.008,0.046\right)\right)$ are statistically significant.

**TABLE 6:** Schizophrenia dataset [28].

	With schizophrenia		Without schizophrenia		Characteristics		
Study	Events	Total	Events	Total	Mean age	% Male	% Diabetes
Barcella et al. [32]	20	984	632	127281	40.3±20.8	48.4	4.6
Tzur Bitan et al. [33]	22	649	7	709	51.5±15.4	60.9	17.1
Fond et al. [34]	4	15	94	1077	63.1±18.5	54.3	23.4
Fond et al. [35]	211	823	10854	49927	70.3±19.2	56.8	27.8
Jeon et al. [36]	6	159	49	2817	55.4±16.2	41.7	15.1
Nemani et al. [37]	20	75	701	6349	54±18.6	47	25.7
Poblador‐Plou et al. [38]	11	40	760	4372	67.7±20.7	41.2	11.9
Rivas‐Ramirez et al. [39]	2	18	3	69	51.5±14.8	47.1	4.5
Rodriguez‐Molinero et al. [40]	2	4	77	414	65.4±16.6	56.9	23.6
Tyson et al. [41]	5	6	70	144	77.6±10.5	50	34

Let the log odds of mortality due to COVID‐19 be the risk measure for the group of schizophrenic patients and for the control group. We consider a classical meta‐analysis model relating the risk measures, and extend it by considering additional covariates represented by the (standardized) mean age, the log odds of male, and the log odds of diabetes. Other covariates reported in the data are not considered because of the presence of too many missing values. Figure S7 in the Supporting Information reports the scatterplots relating the risk measure for schizophrenic patients to each covariate, suggesting the presence of a linear relationship with the risk in the control group, the mean age, and the log odds of diabetes. The extended version of the basic model with only baseline risk information (1) considers the presence of one additional covariate at a time. Since information about within‐study covariances between the additional covariates and the risk measures is unavailable, the pseudo‐likelihood approach (6) is adopted. Results are compared with those from the naive analysis.

Table 7 reports the estimates of the regression coefficients, the residual variance, and the variance for the mismeasured variables obtained from the likelihood approach, the pseudo‐likelihood approach, and the naive analysis. According to all the approaches, there is a significant positive association between the measure of risk for the schizophrenic patients and the measure of risk in the control group, either in the presence or absence of additional covariates. The use of the naive analysis provides smaller values of $\beta_{1}$ than the competing error‐corrected approaches, a result which is quite a common effect of the presence of measurement errors in linear regression models. In addition, the naive analysis gives rise to large values of the residual heterogeneity as a consequence of not taking into account the presence of measurement errors. The inclusion of an additional covariate in the model using a pseudo‐likelihood approach gives rise to interesting results. The mean age of the patients is the only resulting significant covariate, with the indication that the risk of mortality decreases with younger subjects. Such a result is in line with the findings in Pardamean and colleagues [28]. When the covariate is significant, its use in the model provides a substantial reduction of the residual variability.

**TABLE 7:** Estimates of ${\left(\beta_{0},\beta_{1},\beta_{2},\tau^{2}\right)}^{⊤}$ and the associated standard errors when using the likelihood approach to fit the classical model with baseline risk information and the pseudo‐likelihood approach to fit the model with one additional covariate to schizophrenia dataset [28]. Significant coefficients are highlighted.

Covariate	Approach	${\hat{\beta }}_{0}$	${\hat{\beta }}_{1}$	${\hat{\beta }}_{2}$	${\hat{\tau }}^{2}$	${\hat{\sigma }}_{\xi }^{2}$	${\hat{\sigma }}_{\zeta }^{2}$
No	likelihood	0.146 (0.329)	**0.761 (0.091)**	—	0.028 (0.049)	2.459 (1.112)	—
	naive analysis	−0.080 (0.140)	**0.709 (0.056)**	—	0.527 (0.211)	—	—
Scaled mean age	pseudo‐likelihood	0.882 (0.485)	**1.071 (0.185)**	**−0.557 (0.269)**	1.6e‐05 (5e‐04)	2.414 (1.093)	0.922 (0.420)
	naive analysis	0.740 (0.410)	**1.004 (0.149)**	**−0.492 (0.236)**	0.466 (0.174)	—	—
Log odds of male	pseudo‐likelihood	0.102 (0.186)	**0.748 (0.062)**	−0.608 (0.417)	0.003 (0.008)	2.514 (1.164)	0.066 (0.031)
	naive analysis	0.034 (0.177)	**0.724 (0.057)**	−0.422 (0.407)	0.492 (0.184)	—	—
Log odds of diabetes	pseudo‐likelihood	0.195 (0.535)	**0.743 (0.133)**	0.057 (0.274)	0.033 (0.054)	2.578 (1.193)	0.524 (0.255)
	naive analysis	−0.092 (0.195)	**0.718 (0.110)**	−0.025 (0.251)	0.524 (0.196)	—	—

## 6 Conclusions

This paper explores the inclusion of additional study‐specific covariates other than the control rate in meta‐analysis of treatment effectiveness, including baseline risk information, with the aim of better explaining between‐study heterogeneity. Inference is performed through a likelihood approach, accounting for the presence of measurement errors associated with the summarized risk measures and covariate information at the study level. When subgroup study‐specific summary information associated with additional covariates is available, within‐study covariances between the risk measures for cases and controls and the additional covariates are derived using Taylor expansions. When this is not the case, and a fully specified likelihood‐based approach cannot be applied, then a pseudo‐likelihood solution is exploited. The pseudo‐likelihood proposal is defined under a working conditional independence assumption of the observed quantities, thus avoiding within‐study covariances and providing a more robust solution in case of model misspecification with respect to the full likelihood approach. Simulation studies show the applicability of the likelihood and the pseudo‐likelihood proposals under a variety of scenarios, including different specifications for the sample size, the between‐study heterogeneity, and the underlying risk distribution. Results from both the approaches are satisfactory, although the pseudo‐likelihood solution is more appealing in case of deviations from model conditions and it results in less computational effort under an approximate normally distributed measurement error specification.

While the study has been conducted from a inferential frequentist point of view, the use of Bayesian solutions can represent an interesting future extension, especially when the number of additional covariates increases. Specifically, noninformative priors might usefully be assumed for the mean and the variance of the study‐specific additional covariates.

Another line of future research includes the application of functional approaches to model the role of baseline risk and additional study‐level covariates in meta‐analysis of treatment effect, as an alternative to the likelihood solutions. One possibility is represented by SIMEX [12], a simulation extrapolation approach widely used in the literature to correct for the presence of measurement errors without assumptions on the marginal distribution of the underlying error‐prone variables. A first application of SIMEX to model the role of baseline risk is in Guolo [16]. The extension to include additional study‐specific covariates, while being of interest for robustness properties, can be challenging as a consequence of computationally intensive simulation procedures.

As suggested by a reviewer, a further extension of the model might consider an interaction between the baseline risk measure and the additional covariate. This extension can be complex, even computationally, due to the interaction that would be created between the measurement errors associated with the variables.

## Disclosure

The authors have nothing to report.

## Conflicts of Interest

The authors declare no conflicts of interest.

## Supporting information




**Data S1**: Supporting Information.

## Data Availability

Data sharing is not applicable to this article as no datasets were generated or analyzed during the current study.

## Appendix A Formula of the Sandwich Covariance Matrix

The sandwich estimate of the covariance matrix of the estimator $\hat{\theta }$ of θ [22] is

$$\begin{array}{l} G\left(\hat{\theta }\right)=n^{-1}J^{-1}(\theta )I(\theta )J^{-1}(\theta ){|}_{\theta =\hat{\theta }}, \end{array}$$

where

$$\begin{array}{ll} J\left(\theta \right) & =n^{-1}E\left\{\frac{\partial^{2}\log pL_{i}(\theta )}{\partial \theta \partial \theta^{⊤}}\right\}\;\text{and}\\ & \;I(\theta )=n^{-1}E\left[\frac{\partial \log pL_{i}(\theta )}{\partial \theta }{\left\{\frac{\partial \log pL_{i}(\theta )}{\partial \theta }\right\}}^{⊤}\right], \end{array}$$

$pL_{i}(\theta )$ is the contribution of study i to the pseudo‐likelihood for θ, ∂f(x)/∂x is the derivative of f(x) with respect to x and E(X) is the expectation of X. Matrices J(θ) and I(θ) can be consistently estimated by their empirical counterpart.

## Appendix B Pseudo‐Likelihood Approach Under Exact Measurement Error Models

If the covariate is a log odds and under normality assumptions for $\eta_{i},\xi_{i},\zeta_{i}$, a pseudo‐likelihood function can be derived using model (11), as

$$\begin{array}{ll} pL(\theta ) & \propto \overset{n}{\underset{i=1}{\prod }}\int_{-\infty }^{+\infty }\int_{-\infty }^{+\infty }\int_{-\infty }^{+\infty }{\left\{\text{expit}\left(\eta_{i}\right)\right\}}^{Y_{i}}{\left\{1-\text{expit}\left(\eta_{i}\right)\right\}}^{n_{iT}-Y_{i}}\\ & \times {\left\{\text{expit}\left(\xi_{i}\right)\right\}}^{X_{i}}{\left\{1-\text{expit}\left(\xi_{i}\right)\right\}}^{n_{iC}-X_{i}}{\left\{\text{expit}\left(\zeta_{i}\right)\right\}}^{Z_{i}}\\ & \;{\left\{1-\text{expit}\left(\zeta_{i}\right)\right\}}^{n_{i}-Z_{i}}\\ & \times \frac{1}{\sqrt{\tau^{2}}}\phi \left(\frac{\eta_{i}-\beta_{0}-\beta_{1}\xi_{i}-\beta_{2}\zeta_{i}}{\tau }\right)\frac{1}{\sqrt{\sigma_{\xi }^{2}}}\phi \left(\frac{\xi_{i}-\mu_{\xi }}{\sigma_{\xi }}\right)\\ & \;\frac{1}{\sqrt{\sigma_{\zeta }^{2}}}\phi \left(\frac{\zeta_{i}-\mu_{\zeta }}{\sigma_{\zeta }}\right)d\eta_{i}d\xi_{i}d\zeta_{i}. \end{array}$$

When the covariate is a mean value, a pseudo‐likelihood function can be derived using model (15), as

$$\begin{array}{ll} pL(\theta )\propto \; & \overset{n}{\underset{i=1}{\prod }}\int_{-\infty }^{+\infty }\int_{-\infty }^{+\infty }\int_{-\infty }^{+\infty }{\left\{\text{expit}\left(\eta_{i}\right)\right\}}^{Y_{i}}{\left\{1-\text{expit}\left(\eta_{i}\right)\right\}}^{n_{iT}-Y_{i}}\\ \; & \times {\left\{\text{expit}\left(\xi_{i}\right)\right\}}^{X_{i}}{\left\{1-\text{expit}\left(\xi_{i}\right)\right\}}^{n_{iC}-X_{i}}\\ \; & \times \phi \left(\frac{Z_{i}-\zeta_{i}}{SD_{i}/\sqrt{n_{i}}}\right)\times \frac{1}{\sqrt{\tau^{2}}}\phi \left(\frac{\eta_{i}-\beta_{0}-\beta_{1}\xi_{i}-\beta_{2}\zeta_{i}}{\tau }\right)\\ \; & \times \frac{1}{\sqrt{\sigma_{\xi }^{2}}}\phi \left(\frac{\xi_{i}-\mu_{\xi }}{\sigma_{\xi }}\right)\frac{1}{\sqrt{\sigma_{\zeta }^{2}}}\phi \left(\frac{\zeta_{i}-\mu_{\zeta }}{\sigma_{\zeta }}\right)d\eta_{i}d\xi_{i}d\zeta_{i}. \end{array}$$

## Appendix C Proof of Formula of $s_{\eta_{i},\zeta_{i}}$

### Case With $\zeta_{i}$ as a Log Odds

The within‐study covariance between treatment risk and an additional covariate express a log odds can be estimated using the first‐order Taylor's expansion around the means [20]:

$$\begin{array}{ll} s_{\eta_{i},\zeta_{i}} & =cov\left({\hat{\eta }}_{i},{\hat{\zeta }}_{i}\right)\\ & =cov\left(\log\frac{Y_{i}}{n_{iT}-Y_{i}},\log\frac{Z_{i}}{n_{i}-Z_{i}}\right)\\ & =cov\left(\log Y_{i},\log Z_{i}\right)-cov\{\log\left(n_{iT}-Y_{i}\right),\log Z_{i}\}\\ & \;-cov\{\log Y_{i},\log\left(n_{i}-Z_{i}\right)\}+cov\{\log\left(n_{iT}-Y_{i}\right),\\ & \;\log\left(n_{i}-Z_{i}\right)\}\\ & \approx \frac{1}{E\left(Y_{i}\right)E\left(Z_{i}\right)}cov\left(Y_{i},Z_{i}\right)\\ & \;-\frac{1}{E(n_{iT}-Y_{i})E\left(Z_{i}\right)}cov\left(n_{iT}-Y_{i},Z_{i}\right)\\ & \;-\frac{1}{E\left(Y_{i}\right)E(n_{i}-Z_{i})}cov\left(Y_{i},n_{i}-Z_{i}\right)\\ & \;+\frac{1}{E(n_{iT}-Y_{i})E(n_{i}-Z_{i})}cov\left(n_{iT}-Y_{i},n_{i}-Z_{i}\right), \end{array}$$

where cov denotes “within‐study covariance” or “covariance” given ${\left(\eta_{i},\xi_{i},\zeta_{i}\right)}^{⊤}$. In the above expansion, the expected values of $Y_{i}$ and $Z_{i}$ can be estimated with their observed values. Let $U_{ik}$ and $V_{ik}$, k=1,…,n, denote the indicator of the event and the indicator of the male of subject k in the treatment group of study i, respectively. The within‐study covariance between $Y_{i}$ and $Z_{i}$ can be estimated as

$$\begin{array}{ll} cov\left(Y_{i},Z_{i}\right) & =cov\left(Y_{i},Z_{iT1M}+Z_{iT0M}+Z_{iC1M}+Z_{iC0M}\right)\\ & =cov\left(Y_{i},Z_{iT1M}+Z_{iT0M}\right)\\ & =\overset{n_{iT}}{\underset{j=1}{\sum }}\overset{n_{iT}}{\underset{k=1}{\sum }}cov\left(U_{ij},V_{ik}\right)\\ & =\overset{n_{iT}}{\underset{k=1}{\sum }}cov\left(U_{ik},V_{ik}\right)\\ & =n_{iT}cov\left(U_{i1},V_{i1}\right)\\ & =n_{iT}\left\{E(U_{i1}V_{i1})-E(U_{i1})E(V_{i1})\right\}\\ & =n_{iT}\{Pr\left(\text{a treated patient is male and with event}\right)\;\\ & -Pr(\text{a treated patient is with event})\\ & \;\;Pr(\text{a treated patient is male})\}\\ & \approx n_{iT}\left\{\frac{Z_{iT1M}}{n_{iT}}-\frac{Y_{i}}{n_{iT}}\times \frac{\left(Z_{iT1M}+Z_{iT0M}\right)}{n_{iT}}\right\}\\ & =Z_{iT1M}-\frac{Y_{i}\left(Z_{iT1M}+Z_{iT0M}\right)}{n_{iT}}. \end{array}$$

Other within‐study covariances in the expansion of $s_{\eta_{i},\zeta_{i}}$ can be estimated similarly,

$$\begin{array}{ll} cov\left(n_{iT}-Y_{i},Z_{i}\right) & \approx Z_{iT0M}-\frac{(n_{iT}-Y_{i})\left(Z_{iT1M}+Z_{iT0M}\right)}{n_{iT}},\\ cov\left(Y_{i},n_{i}-Z_{i}\right) & \approx Z_{iT1F}-\frac{Y_{i}\left(Z_{iT1F}+Z_{iT0F}\right)}{n_{iT}},\\ cov\left(n_{iT}-Y_{i},n_{i}-Z_{i}\right) & \approx Z_{iT0F}-\frac{(n_{iT}-Y_{i})\left(Z_{iT1F}+Z_{iT0F}\right)}{n_{iT}}. \end{array}$$

Hence, the within‐study covariance between ${\hat{\eta }}_{i}$ and ${\hat{\zeta }}_{i}$ is

$$\begin{array}{lll} s_{\eta_{i},\zeta_{i}} & \approx \; & \frac{1}{Z_{i}}\left(\frac{Z_{iT1M}}{Y_{i}}-\frac{Z_{iT0M}}{n_{iT}-Y_{i}}\right)-\frac{1}{n_{i}-Z_{i}}\left(\frac{Z_{iT1F}}{Y_{i}}-\frac{Z_{iT0F}}{n_{iT}-Y_{i}}\right). \end{array}$$

### Case With $\zeta_{i}$ as a Mean Value

The within‐study covariance between the treatment risk and an additional covariate expressed as a mean value can be approximated using the first‐order Taylor's expansion around the means [20]. Let $Z_{iT}$ and $Z_{iC}$ denote mean values in the treatment group and in the control group, respectively. Thus,

$$\begin{array}{ll} s_{\eta_{i},\zeta_{i}} & =cov\left({\hat{\eta }}_{i},{\hat{\zeta }}_{i}\right)\\ & =cov\left(\log\frac{Y_{i}}{n_{iT}-Y_{i}},Z_{i}\right)\\ & =cov\left(\log\frac{Y_{i}}{n_{iT}-Y_{i}},\frac{n_{iT}Z_{iT}+n_{iC}Z_{iC}}{n_{i}}\right)\\ & =cov\left\{\log Y_{i}-\log(n_{iT}-Y_{i}),\frac{n_{iT}}{n_{i}}Z_{iT}\right\}\\ & =\frac{n_{iT}}{n_{i}}cov\left(\log Y_{i},Z_{iT}\right)-\frac{n_{iT}}{n_{i}}cov\{\log\left(n_{iT}-Y_{i}\right),Z_{iT}\}\\ & \approx \frac{n_{iT}}{n_{i}}\frac{1}{E\left(Y_{i}\right)}cov\left(Y_{i},Z_{iT}\right)\\ & \;-\frac{n_{iT}}{n_{i}}\frac{1}{E\left(n_{iT}-Y_{i}\right)}cov\left(n_{iT}-Y_{i},Z_{iT}\right). \end{array}$$

The expected value of $Y_{i}$ can be estimated with its observed value. Let $U_{ik}$ denote the indicator of the event and $V_{ik}$ denote the age of subject k in the treatment group of study i. The covariance between $Y_{i}$ and $Z_{iT}$ can be estimated as

$$\begin{array}{ll} cov\left(Y_{i},Z_{iT}\right) & =\frac{1}{n_{iT}}cov\left(\overset{n_{iT}}{\underset{j=1}{\sum }}U_{ij},\overset{n_{iT}}{\underset{k=1}{\sum }}V_{ik}\right)\\ & =\frac{1}{n_{iT}}\overset{n_{iT}}{\underset{j=1}{\sum }}\overset{n_{iT}}{\underset{k=1}{\sum }}cov\left(U_{ij},V_{ik}\right)\\ & =\frac{1}{n_{iT}}\overset{n_{iT}}{\underset{k=1}{\sum }}cov\left(U_{ik},V_{ik}\right)\\ & =\frac{1}{n_{iT}}n_{iT}cov\left(U_{i1},V_{i1}\right)\\ & =E\left(U_{i1}V_{i1}\right)-E\left(U_{i1}\right)E\left(V_{i1}\right). \end{array}$$

Since $U_{i1}V_{i1}$ is equal to $V_{i1}$ if treated patient 1 is with event and zero otherwise, its expected value can be estimated by $Y_{i}Z_{iT1}/n_{iT}$, so that

$$\begin{array}{ll} cov\left(Y_{i},Z_{iT}\right) & \approx \frac{Y_{i}}{n_{iT}}\left(Z_{iT1}-Z_{iT}\right). \end{array}$$

The within‐study covariance between $n_{iT}-Y_{i}$ and $Z_{iT}$ can be estimated similarly,

$$\begin{array}{ll} cov\left(n_{iT}-Y_{i},Z_{iT}\right) & \approx \frac{n_{iT}-Y_{i}}{n_{iT}}\left(Z_{iT0}-Z_{iT}\right). \end{array}$$

Therefore, the within‐study covariance between ${\hat{\eta }}_{i}$ and ${\hat{\zeta }}_{i}$ is

$$\begin{array}{ll} s_{\eta_{i},\zeta_{i}} & \approx \frac{1}{n_{i}}\left(Z_{iT1}-Z_{iT0}\right). \end{array}$$
