# Supplementary material for: Modeling the Role of Baseline Risk and Additional Study‐Level Covariates in Meta‐Analysis of Treatment Effects
Source: Stat Med. 2025 Oct 23;44(23-24):e70278. doi: 10.1002/sim.70278 (PMC12548021; doi:10.1002/sim.70278)
Supplement: Supplementary file 1 — Data S1: Supporting Information. [file SIM-44-0-s001.pdf]

Online Supporting Materials for Modelling the Role of Baseline Risk and Additional Study-Level Covariates in Meta-analysis of Treatment Effects by Phuc T. Tran and Annamaria Guolo

S.1: Additional simulation results

This web appendix reports a portion of the results of the simulation study carried out to evaluate the performance of likelihood approach against competing approaches in scenario 1 and the performance of pseudo-likelihood approach against competing approaches in scenarios 2 and 3, as described in Section Simulation study of the main manuscript.

S.2: Additional results for data analysis

This web appendix reports forest plots and scatter plots as described in Section Examples of the main manuscript.

S.2.1: Schizophrenia

References

[1] Pardamean E, Roan W, Iskandar KTA, Prayangga R, Hariyanto TI. Mortality from coronavirus disease 2019 (Covid-19) in patients with schizophrenia: A systematic review, meta-analysis and meta-regression. General Hospital Psychiatry. 2022;75:61–67.

Table S1: Bias, standard error (se), standard deviation (sd) of the maximum likelihood estimators of $\left(\beta_0, \beta_1, \beta_2, \mu_\xi, \mu_\zeta, \tau^2, \sigma_\xi^2\right)^\top$ and number of convergent solutions over 1,000 replicates for the uncorrected approach and the likelihood approach when data follow scenario 1. Underlying risk distributed as a skew Normal $SN\,(0, 1, -5)$.

τ^2	par.	appr.	$n=10$				$n=20$			
			bias	se	sd	convergence	bias	se	sd	convergence
0.1	β_0	lik	0.030	0.255	0.317	988	0.037	0.188	0.201	994
		naive	-0.085	0.244	0.283	1000	-0.134	0.172	0.189	1000
	β_1	lik	0.059	0.321	0.392	988	0.049	0.236	0.248	994
		naive	-0.107	0.259	0.319	1000	-0.173	0.170	0.214	1000
	β_2	lik	-0.002	0.137	0.157	988	-0.012	0.080	0.083	994
		naive	0.013	0.147	0.176	1000	0.017	0.095	0.096	1000
	μ_ξ	lik	0.006	0.189	0.211	988	0.025	0.138	0.150	994
	τ^2	lik	-0.055	0.051	0.061	981	-0.041	0.051	0.056	987
		naive	0.072	0.065	0.109	1000	0.149	0.073	0.106	1000
	σ_ξ^2	lik	-0.057	0.177	0.196	988	-0.067	0.129	0.142	994
0.5	β_0	lik	0.083	0.460	0.627	997	0.053	0.319	0.363	1000
		naive	-0.077	0.431	0.579	1000	-0.135	0.303	0.331	1000
	β_1	lik	0.113	0.586	0.796	997	0.084	0.386	0.454	1000
		naive	-0.084	0.449	0.645	1000	-0.162	0.286	0.350	1000
	β_2	lik	-0.022	0.172	0.217	997	-0.021	0.202	0.221	1000
		naive	0.021	0.233	0.257	1000	0.021	0.300	0.292	1000
	μ_ξ	lik	0.010	0.188	0.202	997	0.031	0.138	0.145	1000
	τ^2	lik	-0.240	0.185	0.218	997	-0.124	0.171	0.183	999
		naive	0.055	0.208	0.323	1000	0.142	0.187	0.254	1000
	σ_ξ^2	lik	-0.066	0.174	0.192	997	-0.072	0.129	0.139	1000
1	β_0	lik	0.138	0.650	0.894	998	0.064	0.426	0.488	1000
		naive	-0.043	0.579	0.767	1000	-0.142	0.392	0.455	1000
	β_1	lik	0.182	0.831	1.212	998	0.109	0.522	0.681	1000
		naive	-0.047	0.626	0.872	1000	-0.165	0.388	0.473	1000
	β_2	lik	-0.024	0.245	0.306	998	-0.023	0.237	0.265	1000
		naive	0.035	0.305	0.421	1000	0.029	0.289	0.335	1000
	μ_ξ	lik	0.026	0.184	0.207	998	0.031	0.138	0.142	1000
	τ^2	lik	-0.446	0.346	0.417	995	-0.261	0.304	0.326	1000
		naive	0.085	0.406	0.670	1000	0.118	0.326	0.400	1000
	σ_ξ^2	lik	-0.081	0.167	0.186	998	-0.074	0.128	0.134	1000

Table S2: Bias, standard error (se), standard deviation (sd) of the maximum likelihood estimators of $\left(\beta_0, \beta_1, \beta_2, \mu_\xi, \mu_\zeta, \tau^2, \sigma^2_\xi, \sigma^2_\zeta\right)^\top$ and numbers of convergent solutions over 1,000 replicates for the uncorrected approach, the likelihood approach and the pseudo-likelihood approach when data follow scenario 2 and $\tau^2 = 0.5$. Underlying risk distributed as a standard Normal.

τ^2	par.	appr.	$n = 10$				$n = 20$			
			bias	se	sd	convergence	bias	se	sd	convergence
0.5	β_0	lik	0.005	0.236	0.279	999	-0.006	0.169	0.183	1000
		pseudo-likelihood	0.004	0.237	0.281	1000	-0.006	0.170	0.184	1000
		naive	0.003	0.273	0.342	1000	-0.006	0.189	0.206	1000
	β_1	lik	-0.009	0.286	0.347	999	0.001	0.196	0.208	1000
		pseudo-likelihood	-0.009	0.287	0.347	1000	0.002	0.196	0.208	1000
		naive	-0.059	0.291	0.367	1000	-0.071	0.186	0.221	1000
	β_2	lik	-0.003	0.272	0.339	999	0.001	0.184	0.200	1000
		pseudo-likelihood	-0.004	0.273	0.337	1000	0.002	0.184	0.199	1000
		naive	-0.010	0.306	0.388	1000	0.001	0.195	0.220	1000
	μ_ξ	lik	0.013	0.297	0.323	999	0.002	0.218	0.231	1000
		pseudo-likelihood	0.012	0.298	0.324	1000	0.002	0.219	0.232	1000
	μ_ζ	lik	-0.004	0.291	0.305	999	-0.003	0.216	0.211	1000
		pseudo-likelihood	-0.003	0.293	0.308	1000	-0.002	0.217	0.212	1000
	τ^2	lik	-0.197	0.199	0.242	994	-0.127	0.165	0.175	1000
		pseudo-likelihood	-0.197	0.201	0.240	997	-0.128	0.166	0.175	1000
		naive	0.082	0.218	0.355	1000	0.119	0.180	0.233	1000
	σ^2_ξ	lik	-0.122	0.429	0.452	999	-0.089	0.317	0.304	1000
		pseudo-likelihood	-0.113	0.434	0.459	1000	-0.082	0.320	0.305	1000
	σ^2_ζ	lik	-0.128	0.408	0.441	999	-0.070	0.308	0.317	1000
		pseudo-likelihood	-0.114	0.414	0.449	1000	-0.058	0.311	0.320	1000

Table S3: Bias, standard error (se), standard deviation (sd) of the maximum likelihood estimators of $\left(\beta_0, \beta_1, \beta_2, \mu_\xi, \mu_\zeta, \tau^2, \sigma^2_\xi, \sigma^2_\zeta\right)^\top$ and numbers of convergent solutions over 1,000 replicates for the uncorrected approach, the likelihood approach and the pseudo-likelihood approach when data follow scenario 2 and $\tau^2 = 1$. Underlying risk distributed as a standard Normal.

τ^2	par.	appr.	$n = 10$				$n = 20$			
			bias	se	sd	convergence	bias	se	sd	convergence
1	β_0	lik	-0.002	0.307	0.376	999	-0.002	0.228	0.252	1000
		pseudo-likelihood	-0.003	0.308	0.378	999	-0.002	0.229	0.253	1000
		naive	-0.007	0.367	0.451	1000	-0.004	0.252	0.307	1000
	β_1	lik	0.002	0.368	0.472	999	0.007	0.264	0.299	1000
		pseudo-likelihood	0.003	0.368	0.471	999	0.009	0.264	0.298	1000
		naive	-0.044	0.396	0.514	1000	-0.051	0.253	0.321	1000
	β_2	lik	-0.015	0.343	0.431	999	-0.021	0.246	0.267	1000
		pseudo-likelihood	-0.014	0.343	0.434	999	-0.021	0.246	0.267	1000
		naive	-0.000	0.397	0.495	1000	-0.007	0.260	0.318	1000
	μ_ξ	lik	-0.005	0.295	0.302	999	0.003	0.217	0.229	1000
		pseudo-likelihood	-0.004	0.297	0.303	999	0.002	0.218	0.231	1000
	μ_ζ	lik	-0.002	0.296	0.318	999	-0.001	0.217	0.224	1000
		pseudo-likelihood	-0.002	0.298	0.319	999	-0.002	0.218	0.224	1000
	τ^2	lik	-0.414	0.347	0.398	998	-0.227	0.306	0.330	1000
		pseudo-likelihood	-0.413	0.349	0.397	997	-0.223	0.308	0.334	1000
		naive	-0.029	0.363	0.540	1000	0.142	0.333	0.445	1000
	σ^2_ξ	lik	-0.134	0.424	0.431	999	-0.102	0.314	0.319	1000
		pseudo-likelihood	-0.128	0.427	0.436	999	-0.094	0.316	0.323	1000
	σ^2_ζ	lik	-0.100	0.421	0.433	999	-0.060	0.311	0.309	1000
		pseudo-likelihood	-0.088	0.426	0.440	999	-0.047	0.315	0.313	1000

Table S4: Bias, standard error (se), standard deviation (sd) of the maximum likelihood estimators of $\left(\beta_0, \beta_1, \beta_2, \mu_\xi, \mu_\zeta, \tau^2, \sigma_\xi^2, \sigma_\zeta^2\right)^\top$ and numbers of convergent solutions over 1,000 replicates for the uncorrected approach, the likelihood approach and the pseudo-likelihood approach when data follow scenario 3 and $\tau^2 = 0.5$. Underlying risk distributed as a standard Normal.

τ^2	par.	appr.	$n = 10$				$n = 20$			
			bias	se	sd	convergence	bias	se	sd	convergence
0.5	β_0	lik	0.006	0.233	0.282	998	0.013	0.172	0.193	1000
		pseudo-lik	0.006	0.232	0.282	1000	0.013	0.172	0.194	1000
		naive	0.001	0.265	0.391	1000	0.006	0.190	0.226	1000
	β_1	lik	0.021	0.288	0.341	998	0.019	0.204	0.227	1000
		pseudo-lik	0.020	0.286	0.342	1000	0.018	0.204	0.226	1000
		naive	-0.029	0.291	0.407	1000	-0.075	0.186	0.223	1000
	β_2	lik	-0.013	0.258	0.321	998	-0.019	0.181	0.204	1000
		pseudo-lik	-0.013	0.257	0.320	1000	-0.019	0.182	0.202	1000
		naive	0.012	0.306	0.427	1000	0.010	0.198	0.234	1000
	μ_ξ	lik	0.005	0.298	0.318	998	-0.008	0.220	0.231	1000
		pseudo-lik	0.004	0.299	0.316	1000	-0.008	0.220	0.231	1000
	μ_ζ	lik	0.010	0.295	0.325	998	-0.006	0.217	0.221	1000
		pseudo-lik	0.008	0.295	0.324	1000	-0.006	0.216	0.220	1000
	τ^2	lik	-0.203	0.195	0.235	996	-0.129	0.169	0.180	999
		pseudo-lik	-0.204	0.194	0.237	1000	-0.128	0.170	0.180	1000
		naive	0.126	0.234	0.397	1000	0.130	0.184	0.244	1000
	σ_ξ^2	lik	-0.115	0.432	0.425	998	-0.090	0.324	0.314	1000
		pseudo-lik	-0.113	0.434	0.428	1000	-0.089	0.325	0.315	1000
	σ_ζ^2	lik	-0.082	0.415	0.446	998	-0.043	0.305	0.309	1000
		pseudo-lik	-0.080	0.416	0.447	1000	-0.045	0.305	0.306	1000

Table S5: Bias, standard error (se), standard deviation (sd) of the maximum likelihood estimators of $\left(\beta_0, \beta_1, \beta_2, \mu_\xi, \mu_\zeta, \tau^2, \sigma^2_\xi, \sigma^2_\zeta\right)^\top$ and numbers of convergent solutions over 1,000 replicates for the uncorrected approach, the likelihood approach and the pseudo-likelihood approach when data follow scenario 3 and $\tau^2 = 1$. Underlying risk distributed as a standard Normal.

τ^2	par.	appr.	$n = 10$				$n = 20$			
			bias	se	sd	convergence	bias	se	sd	convergence
1	β_0	lik	-0.014	0.312	0.397	1000	0.003	0.226	0.240	1000
		pseudo-lik	-0.015	0.313	0.393	999	0.004	0.226	0.241	1000
		naive	-0.030	0.370	0.465	1000	0.002	0.250	0.327	1000
	β_1	lik	-0.008	0.378	0.488	1000	-0.017	0.266	0.293	1000
		pseudo-lik	-0.009	0.378	0.485	999	-0.017	0.266	0.293	1000
		naive	-0.057	0.397	0.511	1000	-0.116	0.246	0.325	1000
	β_2	lik	-0.008	0.340	0.450	1000	-0.027	0.241	0.256	1000
		pseudo-lik	-0.009	0.340	0.446	999	-0.026	0.241	0.255	1000
		naive	0.041	0.408	0.519	1000	0.007	0.269	0.352	1000
	μ_ξ	lik	-0.006	0.298	0.325	1000	0.006	0.218	0.226	1000
		pseudo-lik	-0.006	0.298	0.324	999	0.006	0.218	0.226	1000
	μ_ζ	lik	-0.013	0.293	0.316	1000	0.012	0.214	0.220	1000
		pseudo-lik	-0.012	0.293	0.317	999	0.013	0.214	0.220	1000
	τ^2	lik	-0.382	0.355	0.424	1000	-0.252	0.299	0.321	1000
		pseudo-lik	-0.383	0.355	0.422	999	-0.252	0.299	0.322	1000
		naive	-0.029	0.363	0.563	1000	0.159	0.338	0.448	1000
	σ^2_ξ	lik	-0.111	0.436	0.478	1000	-0.108	0.320	0.321	1000
		pseudo-lik	-0.112	0.435	0.475	999	-0.108	0.320	0.318	1000
	σ^2_ζ	lik	-0.096	0.408	0.426	1000	-0.067	0.297	0.314	1000
		pseudo-lik	-0.098	0.407	0.424	999	-0.066	0.298	0.316	1000

Table S6: Bias, standard error (se), standard deviation (sd) of the maximum likelihood estimators of $\left(\beta_0, \beta_1, \beta_2, \mu_\xi, \mu_\zeta, \tau^2, \sigma^2_\xi, \sigma^2_\zeta\right)^\top$ and numbers of convergent solutions over 1,000 replicates for the uncorrected approach, the likelihood approach and the pseudo-likelihood approach when data follow scenario 3 and $\tau^2 = 0.1$. Underlying risk distributed as a skew Normal $SN(0, 1, -5)$.

τ^2	par.	appr.	$n = 10$				$n = 20$			
			bias	se	sd	convergence	bias	se	sd	convergence
0.1	β_0	lik	0.037	0.289	0.325	984	0.031	0.202	0.229	995
		pseudo-lik	0.038	0.288	0.318	991	0.030	0.201	0.228	994
		naive	-0.100	0.256	0.327	1000	-0.127	0.185	0.225	1000
	β_1	lik	0.050	0.355	0.413	985	0.041	0.250	0.272	995
		pseudo-lik	0.051	0.354	0.406	991	0.039	0.250	0.269	993
		naive	-0.130	0.274	0.374	1000	-0.155	0.185	0.247	1000
	β_2	lik	-0.013	0.160	0.192	986	-0.020	0.120	0.131	997
		pseudo-lik	-0.014	0.160	0.192	992	-0.019	0.120	0.132	995
		naive	0.007	0.173	0.232	1000	0.015	0.126	0.156	1000
	μ_ξ	lik	0.023	0.187	0.203	986	0.028	0.137	0.142	997
		pseudo-lik	0.022	0.187	0.203	992	0.028	0.137	0.144	995
	μ_ζ	lik	0.001	0.295	0.308	986	0.003	0.215	0.227	997
		pseudo-lik	0.001	0.295	0.307	992	0.003	0.215	0.227	995
	τ^2	lik	-0.050	0.058	0.060	979	-0.039	0.058	0.058	980
		pseudo-lik	-0.051	0.058	0.060	986	-0.039	0.058	0.057	974
		naive	0.105	0.077	0.134	1000	0.184	0.083	0.117	1000
	σ^2_ξ	lik	-0.068	0.174	0.188	985	-0.069	0.128	0.136	997
		pseudo-lik	-0.069	0.174	0.186	991	-0.070	0.127	0.134	995
	σ^2_ζ	lik	-0.088	0.413	0.428	986	-0.056	0.302	0.308	997
		pseudo-lik	-0.086	0.414	0.431	992	-0.055	0.302	0.308	995

Table S7: Bias, standard error (se), standard deviation (sd) of the maximum likelihood estimators of $\left(\beta_0, \beta_1, \beta_2, \mu_\xi, \mu_\zeta, \tau^2, \sigma^2_\xi, \sigma^2_\zeta\right)^\top$ and numbers of convergent solutions over 1,000 replicates for the uncorrected approach, the likelihood approach and the pseudo-likelihood approach when data follow scenario 3 and $\tau^2 = 0.5$. Underlying risk distributed as a skew Normal $SN(0, 1, -5)$.

τ^2	par.	appr.	$n = 10$				$n = 20$			
			bias	se	sd	convergence	bias	se	sd	convergence
0.5	β_0	lik	0.086	0.473	0.602	999	0.060	0.320	0.353	999
		pseudo-lik	0.086	0.471	0.605	998	0.058	0.318	0.349	999
		naive	-0.078	0.444	0.561	1000	-0.139	0.294	0.378	1000
	β_1	lik	0.118	0.583	0.746	999	0.073	0.387	0.438	999
		pseudo-lik	0.118	0.580	0.738	998	0.070	0.384	0.427	999
		naive	-0.094	0.468	0.604	1000	-0.165	0.293	0.397	1000
	β_2	lik	-0.013	0.256	0.328	999	-0.026	0.185	0.214	999
		pseudo-lik	-0.013	0.256	0.327	998	-0.026	0.185	0.215	999
		naive	0.016	0.301	0.387	1000	0.027	0.202	0.269	1000
	μ_ξ	lik	0.011	0.185	0.204	999	0.026	0.138	0.139	999
		pseudo-lik	0.011	0.185	0.203	998	0.026	0.138	0.139	999
	μ_ζ	lik	0.026	0.294	0.311	999	-0.012	0.215	0.218	999
		pseudo-lik	0.027	0.294	0.311	998	-0.011	0.215	0.218	1000
	τ^2	lik	-0.217	0.195	0.234	999	-0.152	0.176	0.190	997
		pseudo-lik	-0.218	0.196	0.233	994	-0.153	0.175	0.190	999
		naive	0.041	0.202	0.334	1000	0.202	0.205	0.282	1000
	σ^2_ξ	lik	-0.074	0.171	0.196	999	-0.066	0.130	0.132	999
		pseudo-lik	-0.075	0.171	0.195	998	-0.065	0.130	0.132	999
	σ^2_ζ	lik	-0.092	0.410	0.435	999	-0.054	0.302	0.310	999
		pseudo-lik	-0.090	0.411	0.437	998	-0.054	0.302	0.311	1000

Table S8: Bias, standard error (se), standard deviation (sd) of the maximum likelihood estimators of $\left(\beta_0, \beta_1, \beta_2, \mu_\xi, \mu_\zeta, \tau^2, \sigma^2_\xi, \sigma^2_\zeta\right)^\top$ and numbers of convergent solutions over 1,000 replicates for the uncorrected approach, the likelihood approach and the pseudo-likelihood approach when data follow scenario 3 and $\tau^2 = 1$. Underlying risk distributed as a skew Normal $SN(0, 1, -5)$.

τ^2	par.	appr.	$n = 10$				$n = 20$			
			bias	se	sd	convergence	bias	se	sd	convergence
1	β_0	lik	0.066	0.617	0.786	999	0.080	0.422	0.462	1000
		pseudo-lik	0.075	0.628	0.796	999	0.082	0.423	0.462	1000
		naive	-0.129	0.600	0.732	1000	-0.116	0.395	0.492	1000
	β_1	lik	0.082	0.755	0.987	999	0.085	0.498	0.537	1000
		pseudo-lik	0.095	0.773	1.004	999	0.088	0.499	0.536	1000
		naive	-0.140	0.635	0.790	1000	-0.131	0.394	0.516	1000
	β_2	lik	-0.008	0.345	0.433	1000	-0.051	0.239	0.258	1000
		pseudo-lik	-0.008	0.346	0.432	999	-0.051	0.239	0.259	1000
		naive	0.023	0.416	0.501	1000	0.005	0.266	0.325	1000
	μ_ξ	lik	0.019	0.183	0.203	1000	0.023	0.137	0.144	1000
		pseudo-lik	0.019	0.183	0.203	999	0.023	0.137	0.144	1000
	μ_ζ	lik	0.008	0.289	0.313	1000	0.003	0.217	0.216	1000
		pseudo-lik	0.008	0.289	0.312	999	0.003	0.217	0.217	1000
	τ^2	lik	-0.401	0.361	0.410	997	-0.297	0.306	0.332	999
		pseudo-lik	-0.399	0.363	0.413	997	-0.297	0.307	0.332	999
		naive	-0.029	0.363	0.559	1000	0.198	0.349	0.461	1000
	σ^2_ξ	lik	-0.082	0.168	0.186	999	-0.072	0.128	0.129	1000
		pseudo-lik	-0.083	0.168	0.185	999	-0.071	0.128	0.130	1000
	σ^2_ζ	lik	-0.125	0.395	0.423	1000	-0.038	0.307	0.310	1000
		pseudo-lik	-0.126	0.394	0.421	999	-0.038	0.307	0.310	1000

Table S9: Bias, standard error (se), standard deviation (sd) of the maximum likelihood estimators of $\left(\beta_0, \beta_1, \beta_2, \mu_\xi, \mu_\zeta, \tau^2, \sigma^2_\xi, \sigma^2_\zeta\right)^\top$ and numbers of convergent solutions over 1,000 replicates for the uncorrected approach, the likelihood approach and the pseudo-likelihood approach when data follow scenario 2 and $\tau^2 = 0.1$. Underlying risk distributed as a skew Normal $SN(0, 1, -5)$.

τ^2	par.	appr.	$n = 10$				$n = 20$			
			bias	se	sd	convergence	bias	se	sd	convergence
0.1	β_0	lik	0.038	3.301	0.341	994	0.040	0.207	0.217	993
		pseudo-likelihood	0.032	0.275	0.332	991	0.036	0.210	0.218	994
		naive	-0.089	0.264	0.300	1000	-0.134	0.186	0.209	1000
	β_1	lik	0.047	4.815	0.449	994	0.061	0.260	0.278	993
		pseudo-likelihood	0.039	0.340	0.426	992	0.060	0.261	0.282	994
		naive	-0.109	0.271	0.329	1000	-0.162	0.183	0.237	1000
	β_2	lik	0.001	0.168	0.195	994	-0.010	0.125	0.134	994
		pseudo-likelihood	0.003	0.172	0.192	993	-0.008	0.127	0.134	996
		naive	0.003	0.177	0.208	1000	-0.004	0.124	0.152	1000
	μ_ξ	lik	0.022	0.190	0.200	994	0.031	0.138	0.150	995
		pseudo-likelihood	0.018	0.191	0.201	993	0.025	0.139	0.149	996
	μ_ζ	lik	0.010	0.298	0.325	994	0.017	0.217	0.214	995
		pseudo-likelihood	0.007	0.300	0.328	993	0.020	0.218	0.218	996
	τ^2	lik	-0.053	0.056	0.060	985	-0.032	0.062	0.061	981
		pseudo-likelihood	-0.054	0.057	0.062	980	-0.037	0.061	0.060	986
		naive	0.086	0.070	0.122	1000	0.201	0.088	0.126	1000
	σ^2_ξ	lik	-0.057	0.179	0.189	993	-0.068	0.131	0.135	994
		pseudo-likelihood	-0.055	0.180	0.189	993	-0.064	0.132	0.140	995
	σ^2_ζ	lik	-0.085	0.429	0.453	994	-0.069	0.313	0.302	995
		pseudo-likelihood	-0.071	0.436	0.466	993	-0.060	0.316	0.303	996

Table S10: Bias, standard error (se), standard deviation (sd) of the maximum likelihood estimators of $\left(\beta_0, \beta_1, \beta_2, \mu_\xi, \mu_\zeta, \tau^2, \sigma^2_\xi, \sigma^2_\zeta\right)^\top$ and numbers of convergent solutions over 1,000 replicates for the uncorrected approach, the likelihood approach and the pseudo-likelihood approach when data follow scenario 2 and $\tau^2 = 0.5$. Underlying risk distributed as a skew Normal $SN(0, 1, -5)$.

τ^2	par.	appr.	$n = 10$				$n = 20$			
			bias	se	sd	convergence	bias	se	sd	convergence
0.5	β_0	lik	0.064	0.449	0.564	998	0.076	0.329	0.387	1000
		pseudo-likelihood	0.059	0.454	0.546	998	0.072	0.332	0.382	999
		naive	-0.112	0.441	0.538	1000	-0.124	0.299	0.357	1000
	β_1	lik	0.101	0.561	0.749	998	0.092	0.402	0.477	1000
		pseudo-likelihood	0.091	0.564	0.690	998	0.089	0.403	0.472	999
		naive	-0.126	0.463	0.588	1000	-0.158	0.296	0.365	1000
	β_2	lik	-0.007	0.267	0.346	1000	-0.017	0.191	0.211	1000
		pseudo-likelihood	-0.007	0.269	0.346	999	-0.016	0.192	0.211	1000
		naive	-0.009	0.304	0.388	1000	-0.012	0.197	0.235	1000
	μ_ξ	lik	0.025	0.188	0.198	1000	0.035	0.137	0.145	1000
		pseudo-likelihood	0.021	0.189	0.199	999	0.030	0.138	0.146	1000
	μ_ζ	lik	-0.005	0.294	0.330	1000	-0.005	0.217	0.219	1000
		pseudo-likelihood	-0.003	0.296	0.331	999	-0.003	0.218	0.222	1000
	τ^2	lik	-0.213	0.197	0.234	996	-0.156	0.176	0.188	999
		pseudo-likelihood	-0.213	0.200	0.235	997	-0.154	0.179	0.191	998
		naive	0.039	0.202	0.328	1000	0.191	0.202	0.265	1000
	σ^2_ξ	lik	-0.065	0.176	0.192	999	-0.079	0.128	0.132	1000
		pseudo-likelihood	-0.062	0.177	0.194	998	-0.075	0.129	0.134	999
	σ^2_ζ	lik	-0.115	0.415	0.418	1000	-0.073	0.311	0.313	1000
		pseudo-likelihood	-0.105	0.420	0.423	999	-0.057	0.316	0.318	1000

Table S11: Bias, standard error (se), standard deviation (sd) of the maximum likelihood estimators of $\left(\beta_0, \beta_1, \beta_2, \mu_\xi, \mu_\zeta, \tau^2, \sigma_\xi^2, \sigma_\zeta^2\right)^\top$ and numbers of convergent solutions over 1,000 replicates for the uncorrected approach, the likelihood approach and the pseudo-likelihood approach when data follow scenario 2 and $\tau^2 = 1$. Underlying risk distributed as a skew Normal $SN(0, 1, -5)$.

τ^2	par.	appr.	$n = 10$				$n = 20$			
			bias	se	sd	convergence	bias	se	sd	convergence
1	β_0	lik	0.051	0.592	0.737	1000	0.115	0.427	0.495	999
		pseudo-likelihood	0.052	0.586	0.744	998	0.115	0.431	0.519	999
		naive	-0.104	0.587	0.709	1000	-0.106	0.386	0.543	1000
	β_1	lik	0.050	0.728	0.937	1000	0.113	0.512	0.605	999
		pseudo-likelihood	0.051	0.704	0.937	998	0.114	0.515	0.647	999
		naive	-0.126	0.615	0.758	1000	-0.150	0.384	0.548	1000
	β_2	lik	-0.014	0.346	0.436	1000	-0.029	0.248	0.277	1000
		pseudo-likelihood	-0.016	0.346	0.433	1000	-0.030	0.248	0.275	1000
		naive	-0.005	0.406	0.471	1000	-0.025	0.259	0.374	1000
	μ_ξ	lik	0.014	0.189	0.201	1000	0.033	0.138	0.141	1000
		pseudo-likelihood	0.010	0.190	0.202	1000	0.028	0.139	0.141	1000
	μ_ζ	lik	0.003	0.297	0.321	1000	-0.004	0.218	0.220	1000
		pseudo-likelihood	0.004	0.298	0.322	1000	-0.003	0.220	0.222	1000
	τ^2	lik	-0.434	0.346	0.401	999	-0.298	0.309	0.324	1000
		pseudo-likelihood	-0.432	0.348	0.406	998	-0.297	0.311	0.328	1000
		naive	-0.044	0.358	0.586	1000	0.246	0.363	0.497	1000
	σ_ξ^2	lik	-0.061	0.179	0.191	1000	-0.072	0.130	0.137	999
		pseudo-likelihood	-0.059	0.179	0.191	999	-0.069	0.132	0.138	999
	σ_ζ^2	lik	-0.095	0.424	0.440	1000	-0.056	0.317	0.321	1000
		pseudo-likelihood	-0.085	0.428	0.448	1000	-0.042	0.321	0.324	1000

Table S12: Bias, standard error (se), standard deviation (sd) of the maximum likelihood estimators of $\left(\beta_0, \beta_1, \beta_2, \mu_\xi, \mu_\zeta, \tau^2, \sigma_\xi^2\right)^\top$ and number of convergent solutions over 1,000 replicates for the uncorrected approach and the likelihood approach when data follow scenario 1 with $\zeta_i \sim Bernoulli\,(0.5)$. Underlying risk distributed as a standard Normal.

τ^2	par.	appr.	$n = 10$				$n = 20$			
			bias	se	sd	convergence	bias	se	sd	convergence
0.1	β_0	lik	0.004	0.152	0.184	989	0.001	0.161	0.170	996
		naive	0.006	0.164	0.215	1000	0.004	0.184	0.199	1000
	β_1	lik	0.010	0.174	0.205	989	0.004	0.124	0.130	996
		naive	-0.048	0.164	0.215	1000	-0.052	0.111	0.138	1000
	β_2	lik	-0.025	0.285	0.349	989	-0.021	0.202	0.214	996
		naive	0.003	0.341	0.398	1000	0.012	0.228	0.246	1000
	μ_ξ	lik	-0.000	0.297	0.323	989	-0.007	0.220	0.221	996
	τ^2	lik	-0.055	0.049	0.060	983	-0.033	0.052	0.059	988
		naive	0.089	0.071	0.120	1000	0.148	0.072	0.105	1000
	σ_ξ^2	lik	-0.120	0.426	0.424	989	-0.068	0.323	0.317	996
0.5	β_0	lik	-0.003	0.294	0.356	999	-0.013	0.238	0.264	1000
		naive	0.006	0.320	0.390	1000	-0.014	0.232	0.291	1000
	β_1	lik	-0.003	0.281	0.328	999	0.014	0.201	0.211	1000
		naive	-0.051	0.289	0.337	1000	-0.049	0.189	0.223	1000
	β_2	lik	-0.031	0.425	0.498	999	-0.019	0.327	0.354	1000
		naive	0.010	0.512	0.595	1000	0.031	0.371	0.413	1000
	μ_ξ	lik	-0.001	0.297	0.319	999	-0.018	0.216	0.233	1000
	τ^2	lik	-0.204	0.188	0.226	998	-0.128	0.165	0.175	1000
		naive	0.042	0.203	0.317	1000	0.125	0.182	0.233	1000
	σ_ξ^2	lik	-0.121	0.427	0.431	999	-0.111	0.310	0.313	1000
1	β_0	lik	0.021	0.439	0.585	1000	-0.003	0.380	0.445	1000
		naive	0.019	0.539	0.631	1000	0.007	0.378	0.485	1000
	β_1	lik	0.024	0.363	0.464	1000	-0.013	0.262	0.290	1000
		naive	-0.014	0.387	0.467	1000	-0.063	0.253	0.319	1000
	β_2	lik	-0.058	0.568	0.740	1000	-0.046	0.461	0.538	1000
		naive	-0.008	0.697	0.828	1000	0.000	0.489	0.614	1000
	μ_ξ	lik	0.007	0.295	0.325	1000	0.003	0.216	0.214	1000
	τ^2	lik	-0.418	0.335	0.403	1000	-0.267	0.292	0.296	1000
		naive	-0.111	0.333	0.522	1000	0.119	0.326	0.400	1000
	σ_ξ^2	lik	-0.137	0.420	0.439	1000	-0.112	0.311	0.319	1000

Table S13: Bias, standard error (se), standard deviation (sd) of the maximum likelihood estimators of $\left(\beta_0, \beta_1, \beta_2, \mu_\xi, \mu_\zeta, \tau^2, \sigma^2_\xi\right)^\top$ and number of convergent solutions over 1,000 replicates for the uncorrected approach and the likelihood approach when data follow scenario 1 with $\zeta_i \sim Bernoulli\,(0.5)$. Underlying risk distributed as a skew Normal $SN\,(0, 1, -5)$.

τ^2	par.	appr.	$n = 10$				$n = 20$			
			bias	se	sd	convergence	bias	se	sd	convergence
0.1	β_0	lik	0.028	0.271	0.341	986	0.045	0.209	0.219	996
		naive	-0.097	0.255	0.309	1000	-0.154	0.208	0.219	1000
	β_1	lik	0.029	0.335	0.461	986	0.068	0.227	0.243	996
		naive	-0.134	0.261	0.326	1000	-0.199	0.166	0.203	1000
	β_2	lik	-0.034	0.277	0.336	986	-0.013	0.194	0.212	996
		naive	-0.012	0.319	0.365	1000	0.010	0.217	0.243	1000
	μ_ξ	lik	0.016	0.184	0.205	986	0.019	0.138	0.142	996
	τ^2	lik	-0.050	0.052	0.063	976	-0.034	0.052	0.059	993
		naive	0.077	0.066	0.111	1000	0.127	0.066	0.095	1000
	σ^2_ξ	lik	-0.076	0.167	0.190	986	-0.063	0.129	0.138	996
0.5	β_0	lik	0.086	0.480	0.648	993	0.100	0.408	0.456	1000
		naive	-0.101	0.478	0.541	1000	-0.134	0.418	0.491	1000
	β_1	lik	0.142	0.564	0.822	993	0.129	0.389	0.448	1000
		naive	-0.104	0.466	0.558	1000	-0.177	0.284	0.345	1000
	β_2	lik	-0.006	0.430	0.516	996	-0.028	0.361	0.392	1000
		naive	0.035	0.509	0.569	1000	0.013	0.417	0.487	1000
	μ_ξ	lik	0.025	0.182	0.200	996	0.031	0.137	0.145	1000
	τ^2	lik	-0.216	0.188	0.221	993	-0.120	0.174	0.192	1000
		naive	0.007	0.190	0.303	1000	0.121	0.181	0.233	1000
	σ^2_ξ	lik	-0.086	0.164	0.184	994	-0.075	0.127	0.134	1000
1	β_0	lik	0.052	0.592	0.767	997	0.088	0.503	0.573	1000
		naive	-0.107	0.587	0.741	1000	-0.153	0.516	0.549	1000
	β_1	lik	0.081	0.732	1.008	997	0.100	0.505	0.590	1000
		naive	-0.127	0.635	0.807	1000	-0.189	0.375	0.455	1000
	β_2	lik	-0.035	0.932	1.218	999	-0.057	0.445	0.461	1000
		naive	0.032	0.865	1.324	1000	0.014	0.518	0.536	1000
	μ_ξ	lik	0.018	0.186	0.202	999	0.024	0.138	0.146	1000
	τ^2	lik	-0.405	0.350	0.419	997	-0.248	0.305	0.321	1000
		naive	-0.061	0.351	0.546	1000	0.074	0.313	0.402	1000
	σ^2_ξ	lik	-0.071	0.173	0.196	999	-0.073	0.128	0.143	1000

Table S14: Bias, standard error (se), standard deviation (sd) of the maximum likelihood estimators of $\left(\beta_0, \beta_1, \beta_2, \mu_\xi, \mu_\zeta, \tau^2, \sigma^2_\xi, \sigma^2_\zeta\right)^\top$, empirical coverage probabilities of 95% Wald-type confidence intervals for $(\beta_0, \beta_1, \beta_2, \mu_\xi, \mu_\zeta)^\top$ and number of convergent solutions over 100 replicates for the uncorrected approach, the likelihood approach and the pseudo-likelihood approach assuming the exact measurement error model. Data follow scenario 2 and $(n, \tau^2)^\top = (10, 0.5)^\top$. Underlying risk distributed as a standard Normal.

parameter	measurement error model	bias	se	sd	ecp	convergence
β_0	no	0.003	0.273	0.342	0.856	100
	exact	-0.009	0.233	0.262	0.82	100
	approx.	-0.011	0.238	0.249	0.88	100
β_1	no	-0.059	0.291	0.367	0.827	100
	exact	-0.010	0.266	0.319	0.83	100
	approx.	-0.016	0.280	0.314	0.88	100
β_2	no	-0.010	0.306	0.388	0.847	100
	exact	0.002	0.264	0.360	0.81	100
	approx.	-0.016	0.271	0.354	0.87	100
μ_ξ	exact	0.020	0.309	0.312	0.92	100
	approx.	0.027	0.300	0.300	0.94	100
μ_ζ	exact	-0.084	0.300	0.322	0.89	100
	approx.	-0.087	0.296	0.319	0.90	100
τ^2	no	0.082	0.218	0.355	-	100
	exact	-0.178	0.205	0.243	-	100
	approx.	-0.196	0.200	0.227		98
σ^2_ξ	exact	-0.041	0.474	0.528	-	100
	approx.	-0.103	0.440	0.455		100
σ^2_ζ	exact	-0.058	0.444	0.519	-	100
	approx.	-0.091	0.424	0.477		100

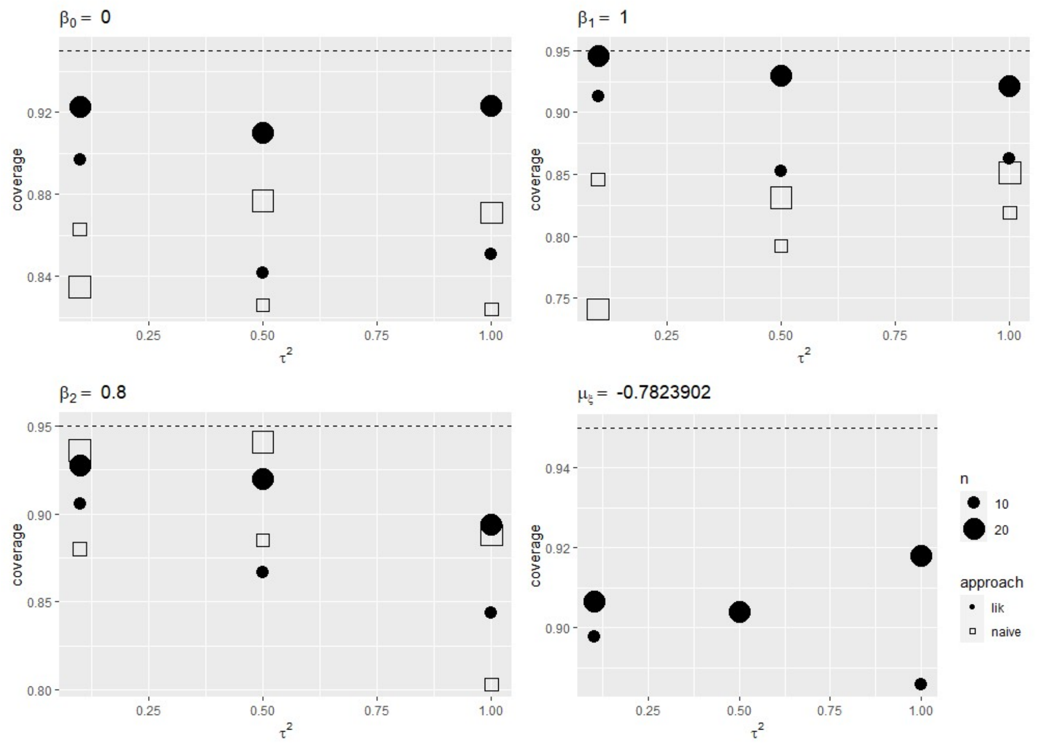

Figure S1: Empirical coverage probabilities of 95% Wald-type confidence intervals for $(\beta_0, \beta_1, \beta_2, \mu_\xi)^\top$ for the uncorrected approach and the likelihood approach when data follow scenario 1. Underlying risk distributed as a skew Normal $SN(0, 1, -5)$.

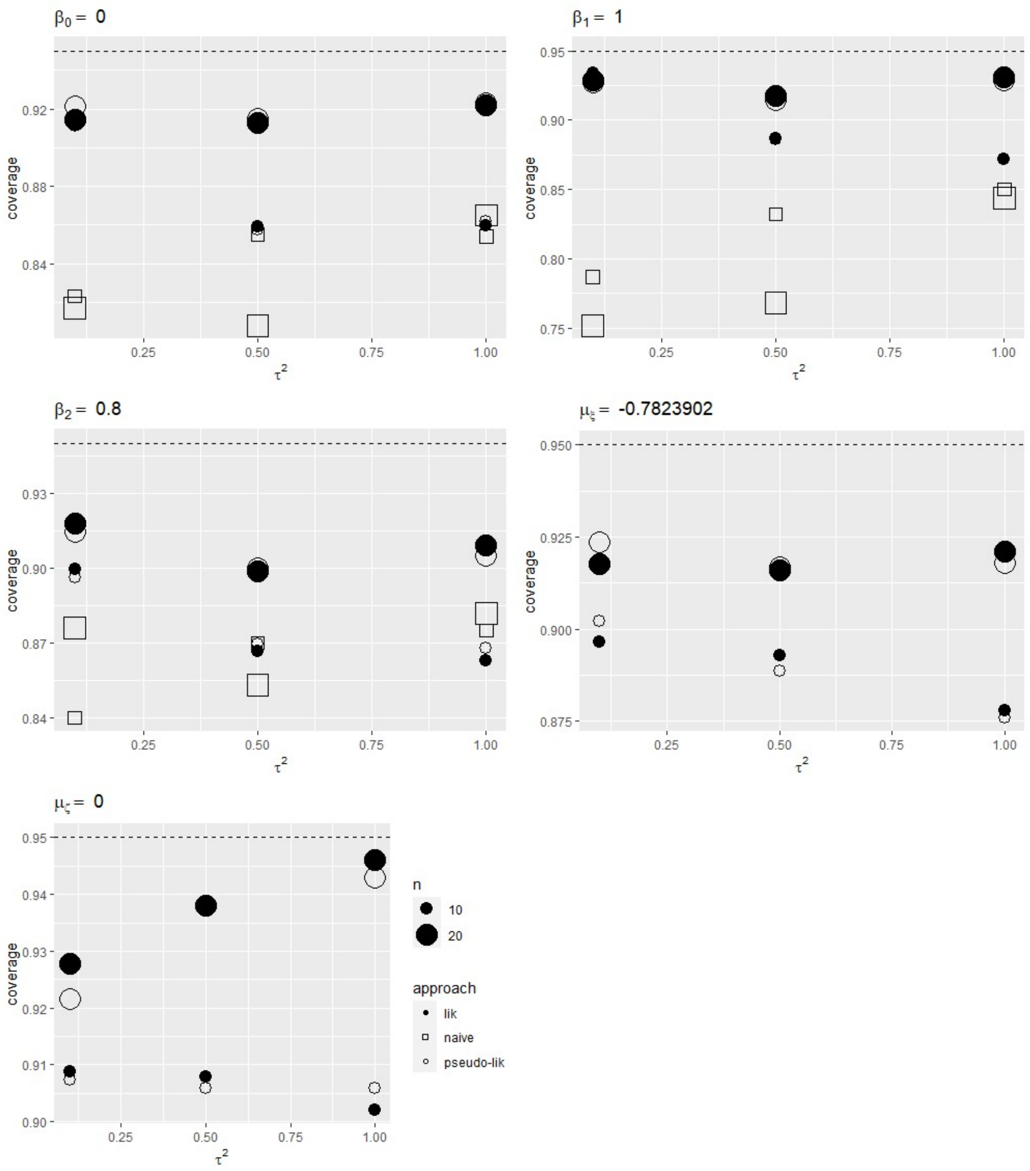

Figure S2: Empirical coverage probabilities of 95% Wald-type confidence intervals for $(\beta_0, \beta_1, \beta_2, \mu_\xi, \mu_\zeta)^\top$ for the uncorrected approach, the likelihood approach and the pseudo-likelihood approach when data follow scenario 3. Underlying risk distributed as a skew Normal $SN(0, 1, -5)$.

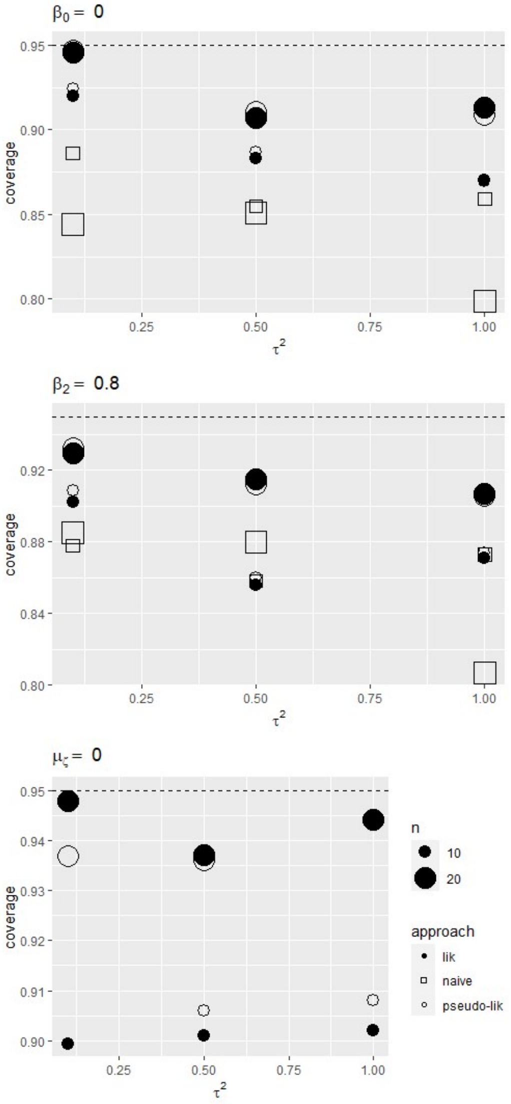

Figure S3: Empirical coverage probabilities of 95% Wald-type confidence intervals for $(\beta_0, \beta_1, \beta_2, \mu_\xi, \mu_\zeta)^\top$ for the uncorrected approach, the likelihood approach and the pseudo-likelihood approach when data follow scenario 2. Underlying risk distributed as a skew Normal $SN(0, 1, -5)$.

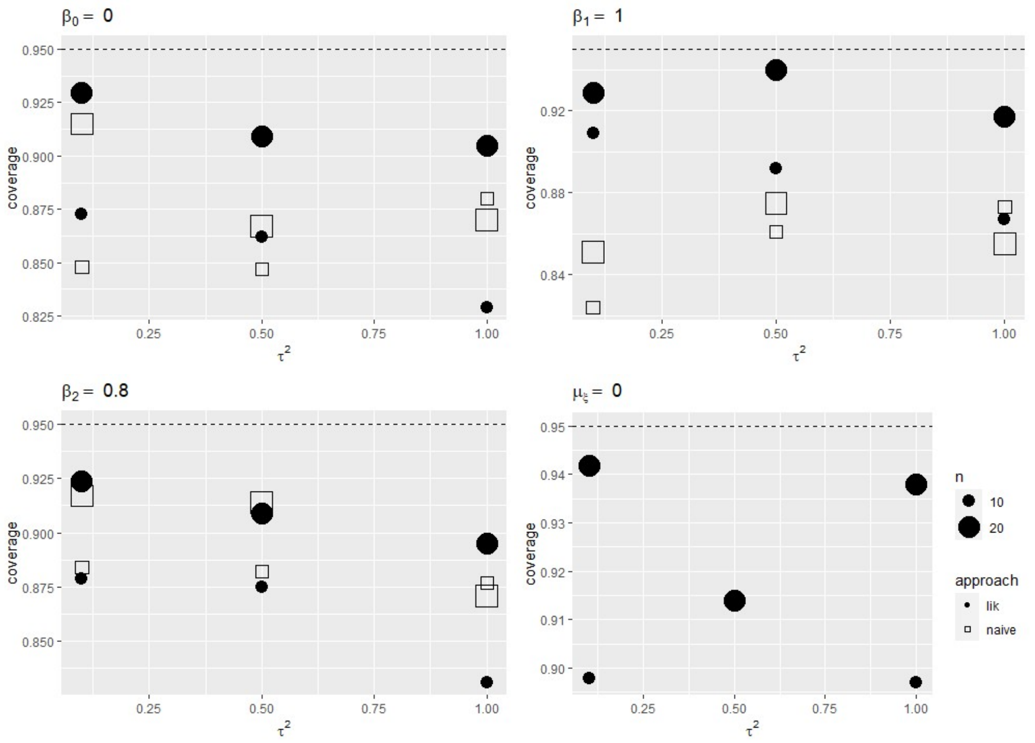

Figure S4: Empirical coverage probabilities of 95% Wald-type confidence intervals for $(\beta_0, \beta_1, \beta_2, \mu_\xi)^\top$ for the uncorrected approach and the likelihood approach when data follow scenario 1 with $\zeta_i \sim \mathit{Bernoulli}\,(0.5)$. Underlying risk distributed as a standard Normal.

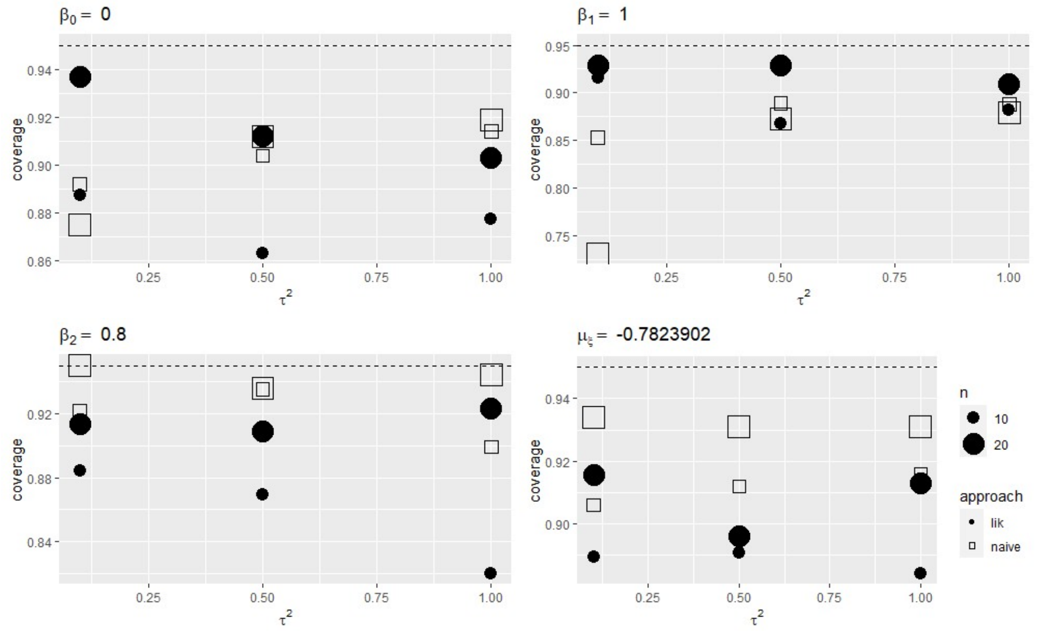

Figure S5: Empirical coverage probabilities of 95% Wald-type confidence intervals for $(\beta_0, \beta_1, \beta_2, \mu_\xi)^\top$ for the uncorrected approach and the likelihood approach when data follow scenario 1 with $\zeta_i \sim Bernoulli\,(0.5)$. Underlying risk distributed as a skew Normal $SN\,(0, 1, -5)$.

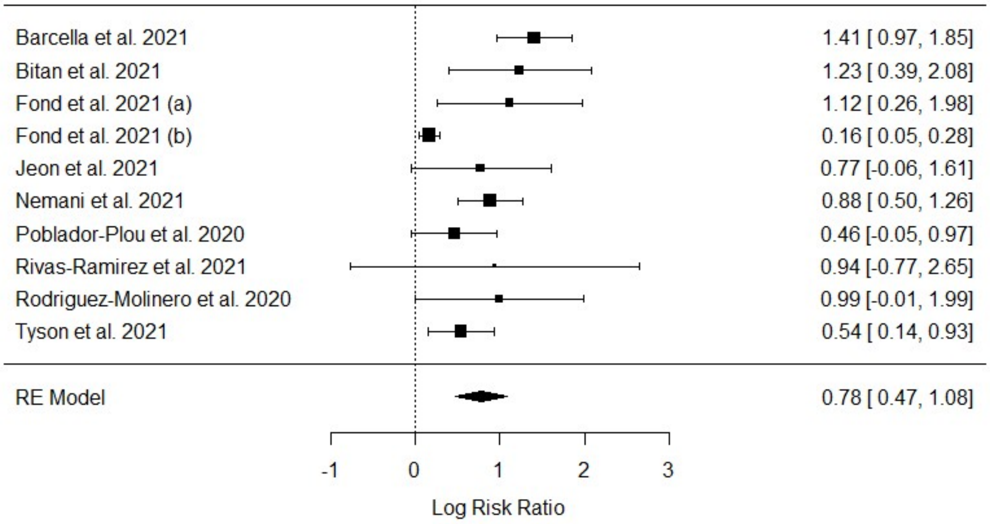

Figure S6: Forest plot for schizophrenia dataset (Pardamean et al., 2022).

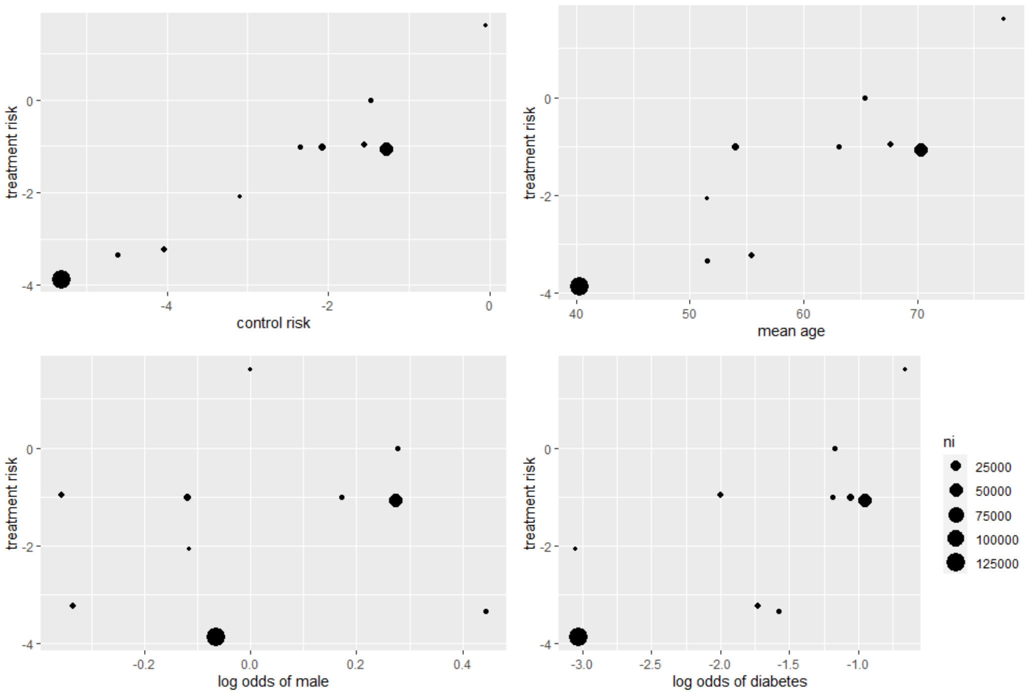

Figure S7: Scatter plots of the treatment risk, the control risk, the mean age, the log odds of male and the log odds of diabetes. Schizophrenia dataset (Pardamean et al., 2022).
